# Cross-species transcriptomic analysis elucidates constitutive aryl hydrocarbon receptor activity

**DOI:** 10.1186/1471-2164-15-1053

**Published:** 2014-12-03

**Authors:** Ren X Sun, Lauren C Chong, Trent T Simmons, Kathleen E Houlahan, Stephenie D Prokopec, John D Watson, Ivy D Moffat, Sanna Lensu, Jere Lindén, Christine P’ng, Allan B Okey, Raimo Pohjanvirta, Paul C Boutros

**Affiliations:** Informatics and Bio-computing Program, Ontario Institute for Cancer Research, Toronto, Canada; Department of Pharmacology & Toxicology, University of Toronto, Toronto, Canada; Department of Environmental Health, National Institute for Health and Welfare, Kuopio, Finland; Department of Biology of Physical Activity, University of Jyväskylä, Jyväskylä, Finland; Department of Veterinary Biosciences, University of Helsinki, Helsinki, Finland; Department of Food Hygiene and Environmental Health, University of Helsinki, Helsinki, Finland; Department of Medical Biophysics, University of Toronto, Toronto, Canada; MaRS Centre, 661 University Avenue, Suite 510, Toronto, M5G 0A3 Ontario, Canada

**Keywords:** Aryl hydrocarbon receptor, AHR endogenous ligands, Constitutive gene expression, TCDD-induced toxicity, Core-gene battery

## Abstract

**Background:**

Research on the aryl hydrocarbon receptor (AHR) has largely focused on variations in toxic outcomes resulting from its activation by halogenated aromatic hydrocarbons. But the AHR also plays key roles in regulating pathways critical for development, and after decades of research the mechanisms underlying physiological regulation by the AHR remain poorly characterized. Previous studies identified several core genes that respond to xenobiotic AHR ligands across a broad range of species and tissues. However, only limited inferences have been made regarding its role in regulating constitutive gene activity, *i.e.* in the absence of exogenous ligands. To address this, we profiled transcriptomic variations between AHR-active and AHR-less-active animals in the absence of an exogenous agonist across five tissues, three of which came from rats (hypothalamus, white adipose and liver) and two of which came from mice (kidney and liver). Because AHR status alone has been shown sufficient to alter transcriptomic responses, we reason that by contrasting profiles amongst AHR-variant animals, we may elucidate effects of the AHR on constitutive mRNA abundances.

**Results:**

We found significantly more overlap in constitutive mRNA abundances amongst tissues within the same species than from tissues between species and identified 13 genes (*Agt*, *Car3*, *Creg1*, *Ctsc*, *E2f6*, *Enpp1*, *Gatm*, *Gstm4*, *Kcnj8*, *Me1*, *Pdk1*, *Slc35a3*, and *Sqrdl*) that are affected by AHR-status in four of five tissues. One gene, *Creg1*, was significantly up-regulated in all AHR-less-active animals. We also find greater overlap between tissues at the pathway level than at the gene level, suggesting coherency to the AHR signalling response within these processes. Analysis of regulatory motifs suggests that the AHR mostly mediates transcriptional regulation via direct binding to response elements.

**Conclusions:**

These findings, though preliminary, present a platform for further evaluating the role of the AHR in regulation of constitutive mRNA levels and physiologic function.

**Electronic supplementary material:**

The online version of this article (doi:10.1186/1471-2164-15-1053) contains supplementary material, which is available to authorized users.

## Background

The aryl hydrocarbon receptor (AHR) is an evolutionarily-conserved, ligand-activated transcription factor and a member of the basic helix-loop-helix/PER-ARNT-SIM family [1]. Proteins within this family participate in signalling and metabolic pathways important for the regulation of circadian rhythm, development and responses to hypoxia and xenobiotic stress [2]. The involvement of the AHR in development has been elucidated on several levels, perhaps most convincingly by studies using transgenic mice, which maintain abolished or considerably-reduced AHR activity [3–7]. *Ahr*^-/-^ mice exhibit a broad range of behavioural, morphological and functional abnormalities including disrupted oculomotor control, cardiomyopathy, vascular hypertrophy, gastric hyperplasia, immune deficiency and reproductive difficulties [3, 4, 7–9]. Recent studies reveal that the AHR also has functional importance in processes such as regulatory T-cell differentiation, cell cycle regulation, mediation of stress responses, inflammation and participation in molecular cross-talk [10–15]. The AHR may even be pathogenically involved in diseases such as hypertension, type II diabetes and cancer [16–19].

The AHR is often described as an environmental sensor for its ability to bind a wide range of compounds and mobilize functionally-relevant gene batteries [20–22]. The classical activation pathway is initiated by ligand-binding, and followed by receptor hetero-dimerization, entry into the nucleus and interactions with regulatory regions of target genes to alter their transcript abundance [23]. By virtue of this ligand imperative, it is probable that the AHR relies on endogenous activators to carry out its developmental responsibilities. Identification of nuclear AHR complexes in cells and tissues not treated with xenobiotics has provided molecular evidence for the existence of such endogenous ligands [24, 25]. Similarly, indole-containing compounds from dietary sources have been shown to undergo metabolism to higher-affinity AHR agonists in mammalian digestive tracts [26, 27]. Many endogenous candidates have been proposed. Notable examples include tryptophan derivatives, arachidonic acid metabolites, 7-ketocholestrol and carotinoids, all of which exhibit AHR binding as well as participation in processes coherent with existing knowledge of AHR function [28–32]. Although there are numerous candidate endogenous AHR ligands, our understanding of their physiologic roles remains incomplete.

In contrast, exogenous AHR ligands have been studied much more extensively. While exogenous ligands as well as endogenous ligands exhibit an exceptional range of structures and biological activities, exogenous ligands generally exhibit AHR binding affinities that are considerably greater than that of their endogenous counterparts [33]. The most potent of these is 2,3,7,8-tetrachlorodibenzo-*p*-dioxin (TCDD). TCDD is a widespread environmental toxicant formed as a by-product of industrial processes involving thermal reactions, herbicide production and low-temperature waste incineration [23]. Exposure in laboratory animals has led to a wide range of toxicological endpoints, yet these vary drastically across species. For example, guinea pigs are extremely sensitive (LD_50_ 1–2 μg/kg), while hamsters are highly resistant (LD_50_ 1000–5000 μg/kg) [34]. This variation also exists within species, as shown by studies of Long-Evans (*Turku/AB*; L-E, LD_50_ = 17.7 μg/kg) and Han/Wistar (*Kuopio*; H/W, LD_50_ > 9600 μg/kg) rats [34]. Many investigations have been conducted to evaluate transcriptomic profiles induced by xenobiotics in AHR-active and -less-active animals [35–45], but few have attempted to characterize, *in vivo*, transcriptomic profiles elicited by endogenous ligands. To address this, we exploit two animal models that both present phenotypically-divergent responses to exogenous ligands based on differences in AHR-status: a mouse AHR knockout model and the H/W rat model of reduced sensitivity to toxic effects of TCDD. By contrasting transcriptomic profiles in the absence of xenobiotic treatment, we hope to capture genes correlated with AHR function and elucidate underlying mechanisms of AHR physiology.

## Methods

### Samples and experimental design

We assessed transcriptomic profiles in rat white adipose tissue (from here on referred to as “adipose”) and hypothalamus tissue. We also re-analyzed constitutive mRNA abundance in samples from untreated animals from three experiments [37, 46] to extend our analysis to two species (mouse and rat) and a total of five tissue types, three of which came from rats (hypothalamus, white adipose and liver) and two of which came from mice (kidney and liver). We use the term “AHR-less-active” to described H/W rats and AHR-null C57BL/6J mice and “AHR-active” in reference to L-E rats and C57BL/6J mice which have wild-type AHRs. A more detailed outline of animal characteristics is available in Table 1.

**Table 1:** **Study sample characteristics**

Species	Tissue	AHR-active		AHR-less-active	
		Strain	n	Strain	n
Mouse	Kidney	C57BL/6J (wild-type)	6	C57BL/6J (AHR-null)	3
Mouse	Liver	C57BL/6J (wild-type)	5	C57BL/6J (AHR-null)	3
Rat	Liver	Long-Evans (Turku/AB)	3	Han/Wistar (Kuopio)	4
Rat	Adipose	Long-Evans (Turku/AB)	3	Han/Wistar (Kuopio)	4
Rat	Hypothalamus	Long-Evans (Turku/AB)	4	Han/Wistar (Kuopio)	4

The composition of each study is described by species, tissues, strains, AHR-status and the number of samples for each group.

### Animal handling and tissue preparation

Detailed procedures of animal handling for past experiments have been previously outlined [37, 46]. All study plans were approved by the Animal Experiment Committee of the University of Kuopio and the Provincial Government of Eastern Finland and all animal handling and reporting comply with ARRIVE guidelines [47]. Animals for the current study were bred and housed at the National Public Health Institute, Division of Environmental Health, in Kuopio, Finland. Four male H/W and four male L-E rats were used for each of the adipose and hypothalamus studies. Animals were singly-housed in stainless-steel wire-mesh cages, the housing environment maintaining a 12-hour light/dark cycle (with lights on at 7:00 am) and temperature and relative humidity of 21 ± 1°C and 50 ± 10% respectively. Pelleted feed and tap water were available *ad libitum*.

To maintain consistency with experimental conditions, all rats were administered a single dose of corn oil via oral gavage at 15–16 weeks of age for H/W rats and 18–19 weeks of age for L-E rats to compensate for the accelerated growth rates of the H/W strain. In the hypothalamus cohort, euthanasia by decapitation was performed approximately 23 hours following corn oil administration, near the end of the dark phase (between 5:40 am and 6:45 am). In the adipose cohort, this was done during light hours, approximately 24 hours post corn oil administration. Samples were then rapidly collected, weighed, snap frozen in liquid nitrogen and stored at -80°C or lower until processed. Adipose tissues were harvested from the inguinal region of animals while the hypothalamus was removed using incision sites along the rostral border of the optic chiasm, caudal border of the mamillary body, ventral border of the anterior commissure and lateral borders of the tuber cinereum and mamillary body complexes.

### Sample processing and microarray hybridization

In all experiments, total RNA was extracted using Qiagen RNeasy kits following the manufacturer’s instructions (Qiagen, Mississauga, Canada). Total RNA yield was quantified by UV spectrophotometry and RNA integrity verified using an Agilent 2100 Bioanalyzer (Agilent Technologies, Santa Clara, CA). RNA levels for rat samples were measured on Affymetrix RAE230-2 arrays and those from mouse samples on Affymetrix MOE430-2 arrays (Affymetrix, Santa Clara, CA) at The Centre for Applied Genomics (Toronto, Canada). RNA abundances were quantified using an Affymetrix GeneChip Scanner 3000 following standard manufacturer protocols.

### Data preparation and visualization

Raw data were loaded in the R statistical environment (v3.0.2) and normalized using the RMA algorithm with the affy package (v1.40.0) of the BioConductor open-source project [48]. Probes for the rat and mouse studies were annotated using the rat2302rnentrezgcdf (v18.0.0) and mouse4302mmentrezgcdf (v18.0.0) packages respectively [49]. All data were tested for spatial and distributional homogeneity using unsupervised pattern recognition with the divisive clustering algorithm (DIANA) in the cluster package (v1.14.4) using Pearson’s correlation as the similarity metric (Additional file 1: Figure S1, Additional file 2: Figure S2, Additional file 3: Figure S3, Additional file 4: Figure S4, Additional file 5: Figure S5). One array from the L-E group in the adipose study (RAE2302_083106W_AO07.CEL) was deemed an outlier due to failure to properly normalize (Additional file 4: Figure S4). This array was removed from downstream analysis and the remaining arrays were re-normalized (with n = 3 in the L-E group). Exclusion of the outlier array improved overall spatial and distributional homogeneity (Additional file 6: Figure S6). Data visualization was facilitated by the lattice (v0.20-27), latticeExtra (v0.6-26) and VennDiagram (v1.6.4) packages [50]. All raw and normalized microarray data can be found in the National Center for Biotechnology Information Gene Expression Omnibus archive (http://www.ncbi.nlm.nih.gov/geo/) under the accessions GSE15857 (mouse kidney), GSE15858 (mouse liver), GSE18301 (rat adipose), GSE18257 (rat hypothalamus) and GSE13513 (rat liver).

### Statistical analysis

Statistical analyses of microarray data were performed in the R statistical environment (v3.0.2) using the limma package (v3.18.13) [51]. A linear model was fit to examine potential differences occurring as a result of AHR genotype. The contrast used in all cases was:
1

We hypothesized that the AHR present in the AHR-less-active strains are inherently different from their AHR-active counterparts – with this difference ultimately reflected in the mRNA abundance of genes under AHR regulatory control. Using the general linear model:
2

where *Y*_*gij*_ is the abundance of gene *g* at condition *i* and replicate *j, μ*_*g*_*is* the estimate of the gene effect, *α*_*gi*_ the estimate of AHR-status effect and *ϵ*_*gij*_ the error term, with the goal of capturing the response of any gene *g* (*Y*_*g*_) exclusively as a result of the effect of the AHR (*α*_*AHR*_):
3

An empirical Bayes method was applied following linear modelling to reduce standard error and a moderated t-test was used to identify differential abundance between different AHR genotypes [51]. All *p*-values were adjusted for multiple testing using a 5% false discovery rate [52]. Significance was defined at *q*-value < 0.05 unless stated otherwise. Interspecies comparisons were conducted through HomoloGene IDs (HID). HomoloGene data were obtained from the National Center for Biotechnology Information (NCBI) HomoloGene database (http://www.ncbi.nlm.nih.gov/homologene, accessed on March 21, 2014). HIDs act as a surrogate for comparing gene homologues across eukaryotic species. All Entrez Gene IDs were matched to a corresponding HID where available and duplicates and those failing to annotate were removed from the final analysis. This left a final count of 10,445 genes. Fold changes and *q*-values for all genes in each experiment are available in Additional file 7: Table S1, Additional file 8: Table S2, Additional file 9: Table S3, Additional file 10: Table S4, Additional file 11: Table S5.

Hypergeometric testing was performed on all common genes between studies to assess whether the observed overlap was significantly greater than expected by chance alone. In the context of this test, we defined “enrichment ratio” as:
4

We also employed this test to assess, separately for each study, whether significantly altered genes were biased for specific chromosomes (Additional file 12: Table S6).

To assess significance of overlap amongst three rat tissues, we performed a bootstrap to estimate the *p*-value. For this, we used a total of three variables, each representing one of the three rat tissues. Each variable constituted a vector of positions of length *n* matching the number of significant genes in each rat tissue (*n* = 1,187 for rat liver, *n* = 318 for rat hypothalamus and *n* = 316 for rat adipose). We randomly allocated the positions in each variable vector to a vector of positions that represented the total number of genes in our analysis (*n* = 10,445). We then counted the positions (*i.e.* rows) that were true for all three variables and repeated this process 10^6^ times. We used the distribution thus generated to evaluate the fraction of permuted counts that was greater than our observed overlap and used this to approximate the *p*-value. We have provided the R code used to generate this analysis as an additional (Additional file 13).

We performed differential power analysis to verify that the removal of one array from the L-E group from the adipose study did not reduce our statistical power. For this, we repeated the systematic removal of an array from the H/W group, one at a time, followed by re-normalization and re-fitting of the entire data set (matching the number of samples at each instance in the H/W group with n = 3). Our analysis indicated that results obtained using n = 3 or n = 4 were comparable, thus affirming our decision to move forward in our analyses using all four H/W samples (Additional file 14: Figure S7).

### Transcription-factor binding site analysis

The AHR has been shown to associate with conserved DNA response elements AHRE-I and AHRE-II during transcription [53, 54]. Hence the presence of these elements in the upstream 5′-regulatory region of genes would provide further evidence for AHR involvement in their regulation. We quantified the occurrence (count) and conservation (score) of four motifs for each of our candidate genes using the sequences GCGTG, TNGCGTG, [T|G]NGCGTG[A|C][G|C]A and CATG{N6}C[T|A]TG, representing the AHRE-I (Core), AHRE-I (Extended), AHRE-I (Full) and AHRE-II motifs respectively [53, 54]. Using REFLINK and REFFLAT tables obtained from UCSC genome browser data (mm9, downloaded on May 9, 2012), transcription start sites were determined and parsed ±3 kilo base pairs (kbp) for the aforementioned motifs [55]. A PhyloHMM conservation score was calculated between zero and one to provide a metric for assessing the strength of conservation across species, with a score of 0.0 indicating no conservation, and a score of 1.0 indicating perfect conservation [56]. Distributions of counts and scores for all motifs were compared between sets of significantly altered genes and insignificant genes within each experiment (Additional file 15: Figure S8, Additional file 16: Figure S9, Additional file 17: Figure S10, Additional file 18: Figure S11).

### AHR binding analysis

AHR binding to regulatory motifs was assessed through analyses using a publicly available dataset that studied the AHR using chromatin immunoprecipitation with DNA microarray technology (ChIP-chip) [57]. RMA-normalized data for control samples treated only with DMSO were obtained from the Gene Expression Omnibus (GSE11850, files GSM299302-GSM299307) and reformatted to an appropriate format for analysis (BED files) using a custom script. Genomic regions were annotated for the nearest gene within 1 kb upstream and downstream of the transcription start site using cisGenome [58] and REFFLAT tables (mm7, downloaded on June 2, 2014) from the UCSC genome browser [55]. The output was parsed using a custom script to remove regions that failed to annotate. The final annotated file was loaded into the R statistical environment (v3.1.0) and a student’s t-test was used to identify probes that preferentially bound to the AHR. A liberal approach was taken for multiple probes mapping to the same gene, in which only the probe with the lowest *p*-value was kept. The fraction of AHR binding was then calculated using:
5

### Gene ontology

Analysis of gene ontology (GO) enrichment was performed using the web tool GoMiner (v2011-01) [59]. Genes that were significantly altered by AHR status at *q* < 0.05 were assessed for enriched ontologies using all species-relevant databases, look-up options and gene ontologies. All results were tested against a null distribution generated using 1000 permutations and a false discovery rate threshold of 0.1%, with the minimum category size in all incidences set to five. Significance for GO analysis was defined at FDR <0.01. A significance filter was applied to raw GoMiner outputs and only GO terms meeting the FDR criterion were subsequently analyzed. Using a custom script, we appended a list of changed genes to each GO term. Since the relationship between GO terms mirrors a directed acyclic graph, we used another custom script to trace all significant GO terms back to their parent GO term to reflect higher-order functional grouping. Significant GO terms and their enrichment scores, changed gene lists, associated parent GO term and FDR values are provided in Additional file 19: Table S7, Additional file 20: Table S8, Additional file 21: Table S9, Additional file 22: Table S10.

## Results

To comprehensively study constitutive AHR effects, we measured mRNA abundances in adipose and hypothalamic tissues of H/W and L-E rats in the absence of exogenous ligands. A common consequence of TCDD exposure in laboratory animals is anorexia-like wasting syndrome [60]. Since TCDD toxicity is mediated via the classical AHR activation pathway and the adipose and hypothalamic tissues are major sites within the body for energy storage and feeding regulation, these tissues were selected as proxies to measure constitutive AHR effects [61]. We supplemented our analysis with control animals from three previous experiments that had been conducted by our lab [35, 37, 46] to expand our coverage of species and tissue types across mouse kidney, mouse liver, rat adipose, rat hypothalamus and rat liver.

### Transcriptomic profile of constitutive AHR activation

**Figure 1:**
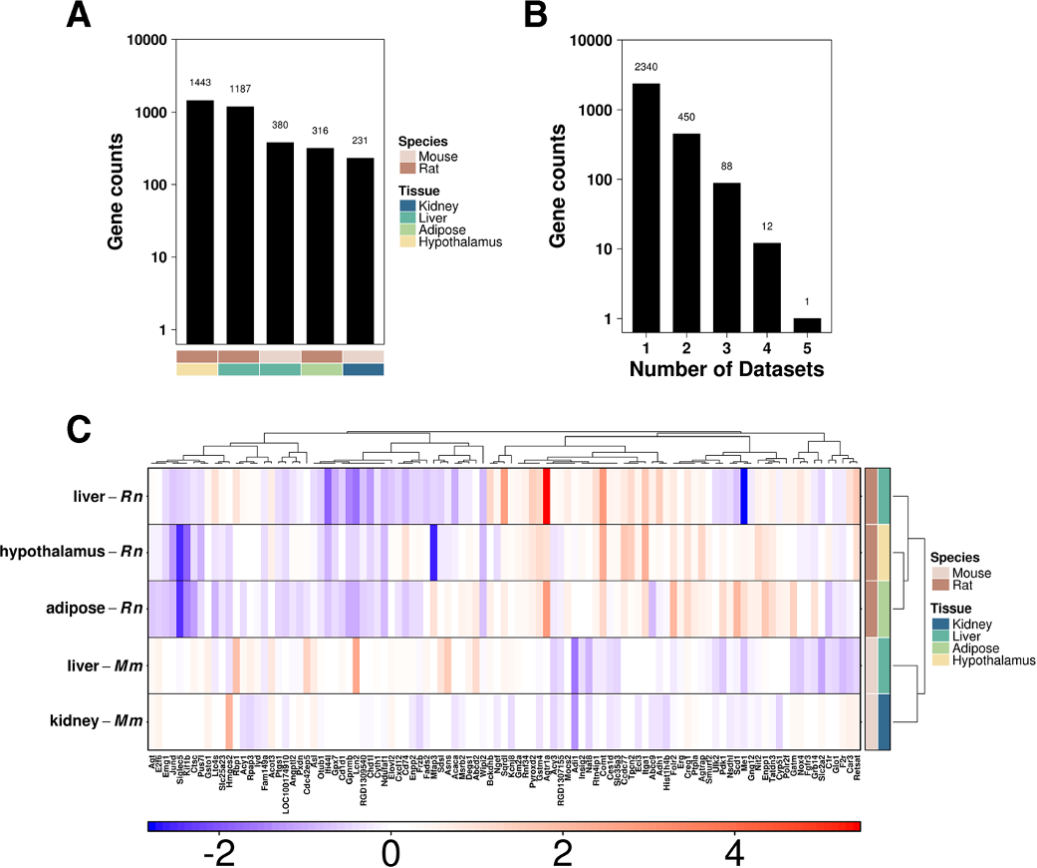
**Overview of significantly altered genes.** The number of genes statistically associated with AHR-status (*q* < 0.05) in each individual tissue **(A)** and across multiple tissues **(B)**. Variations in constitutive mRNA levels were visualized in a heatmap of log_2_ fold changes, using significant genes common to at least two tissues **(C)**.

Our preliminary assessment of data quality demonstrated that our results were not sensitive to the significance threshold (Additional file 23: Figure S12) and similar numbers of genes were up-regulated as down-regulated by the presence of an AHR-active form of the receptor (Additional file 24: Figure S13). We performed within-experiment linear modelling and imposed a significance selection criterion (*q* < 0.05); this revealed a range of 231 genes (mouse, kidney) to 1,443 genes (rat, hypothalamus) whose mRNA levels were differentially-abundant within experiments (Figure 1A). mRNA levels for these genes were also dependent on AHR-status across multiple species and tissues (Figure 1B). Amongst these, 12 genes (*Agt*, *Car3*, *Ctsc*, *E2f6*, *Enpp1*, *Gatm*, *Gstm4*, *Kcnj8*, *Me1*, *Pdk1*, *Slc35a3*, and *Sqrdl*) were significantly altered in four out of five experiments while one gene (*Creg1*) was up-regulated in AHR-less-active animals in all five tissues. Genes differentially-abundant in more than one tissue (n = 101) presented distinct patterns in fold-change magnitudes and directions that clustered tightly with species (Adjusted Rand Index, ARI = 1), not tissue types (ARI = -0.11, Figure 1C).

**Figure 2:**
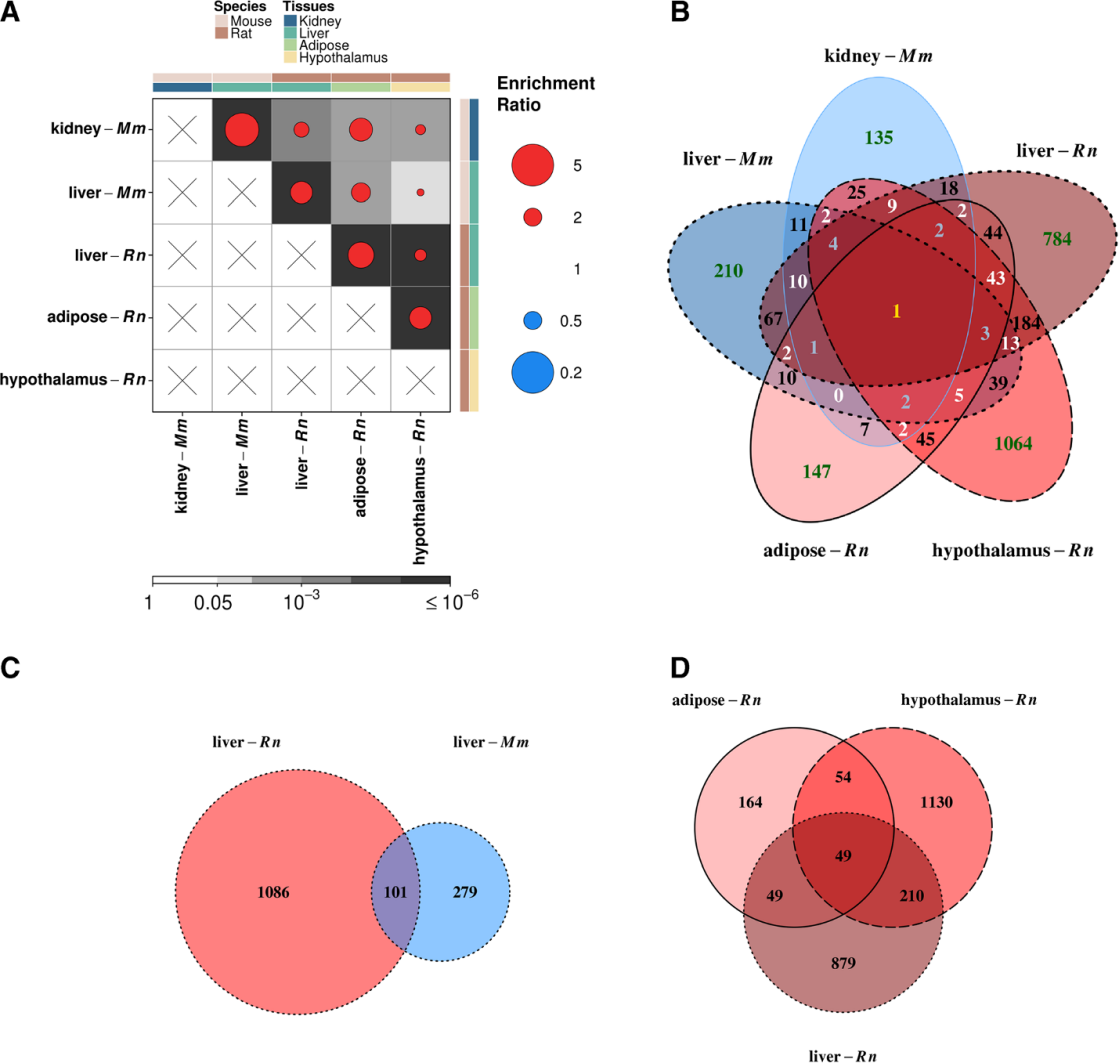
**Assessment of transcriptomic similarity.** Hypergeometric testing was conducted on all pairs of tissues to identify similar transcriptomic profiles **(A)**. Spot size represents the magnitude of the calculated gene enrichment ratio while the background shade denotes *q*-values obtained from hypergeometric testing. Genes not probed on the array are represented by “X”. Venn diagrams of commonly altered genes between all tissues **(B)**, rat tissues **(C)** and liver tissues **(D)**.

To further investigate these potential AHR-regulated core functions, we examined genes altered in multiple species and tissues and performed hypergeometric testing to establish if some tissue/species pairs shared similar AHR-associates of transcriptomic profile. The enrichment ratios computed for commonly altered genes confirm our earlier statement that there was a distinct species-driven pattern in AHR-mediated signalling, with a milder pattern exhibited across the same tissues (Figure 2A, raw counts of gene overlaps are available in Additional file 25: Figure S14). For example, rat liver and rat adipose had 2.7 times more significant genes in common than expected by chance (observed = 98, expected = 36, *q* = 2.01^-20^, Figure 2B). This trend was also observed for mouse kidney and mouse liver where the overlap was 3.7 times more than the expected (observed = 31, expected = 9, *q* = 1.05 × 10^-9^). Alternatively, a more moderate association was observed between mouse and rat liver, despite organ homology, and an enrichment ratio of 2.3 was noted (observed = 101, expected = 44, *q* = 2.39 × 10^-16^, Figure 2C). Pairwise assessments of all overlaps are available in Additional file 26: Figure S15. A set of 49 genes were commonly altered across all rat tissues examined (Figure 2D). From our bootstrap test employing 10^6^ permutations, we estimated the significance of this overlap to be *p* < 10^-6^. Of these, six genes (*Creg1*, Ctsc, *Enpp1*, *Gstm4 Pdk1* and *Sqrdl*) were detected in mouse tissues, suggesting the other 43 genes may contribute to rat-specific events.

### A set of proposed endogenously-regulated AHR core genes

**Figure 3:**
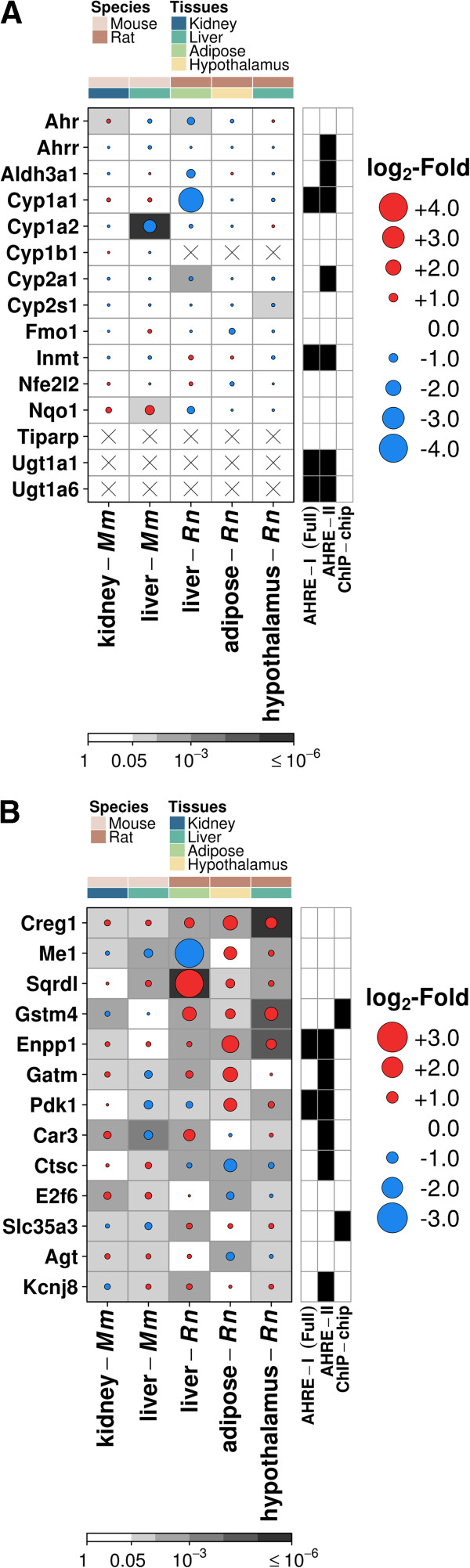
**Transcriptomic profiles of exogenous and endogenous AHR activation.** Transcriptional profiling of AHR core-response genes shows little association with AHR-status in the absence of exogenous ligands **(A)**. By contrast, 13 genes were observed to significantly differ based on AHR-status between animals in at least four tissues, with most of these demonstrating higher levels in AHR-less-active animals **(B)**. All fold changes shown are in log_2_ scale, with the magnitude represented by spot size and *q*-values are denoted by background shade. Only rat gene names are shown. Genes are ordered alphabetically for AHR core-response genes **(A)** and by decreasing average absolute magnitude of change across studies for AHR constitutive genes **(B)**.

To compare ligand-induced and constitutive profiles of well-known AHR target genes, we studied a set of genes, called the AHR core-response genes, previously confirmed to be altered by TCDD via the classical AHR activation pathway in a wide range of tissues: *Ahrr*, *Aldh3a1*, *Cyp1a1*, *Cyp1a2*, *Cyp1b1*, *Cyp2a1*, *Fmo1*, *Inmt*, *Nfe2l2*, *Nqo1*, *Tiparp* and *Ugt1a1* (or *Ugt1a6*) [21, 35, 62–64]. We included the abundance of *Ahr* as a reference to facilitate biological interpretation. Abundance of most of the AHR core-response genes were not significantly altered in the absence of an exogenous ligand (Figure 3A). Interestingly though, AHR-less-active animals appear to exhibit lower mRNA levels for these genes compared to their AHR-active counterparts without xenobiotic treatment (*p* = 0.00026, 95% confidence interval: 0.63-1.00, one-sample prop test), correlated with our assumption of lower AHR activity in these animals. It is also worth noting that although most of the AHR core genes possess binding motifs AHRE-I (Full) and AHRE-II, none were found to associate with AHR binding in the absence of xenobiotics [57].

Next, we focused on genes whose abundance was significantly dependent on AHR-status in at least four out of our five studies (n = 13). Of these, *Creg1* was up-regulated in AHR-less-active animals across all species and tissues (M = 0.39 – 1.3, *q* = 6.07 × 10^-6^ – 0.02, Figure 3B). In contrast to patterns displayed by AHR core-response genes, most of our constitutive candidates appear to exhibit up-regulatory trends in the AHR-less-active animals (*p* = 0.0014, 95% confidence interval: 0.58-1.00, one-sample prop test). Overall, three genes (*Creg1*, *Enpp1* and *Sqrdl*) showed consistent up-regulation across tissues and species, three genes (*Ctsc*, *Gstm4* and *Slc35a3*) displayed diverging species-dependent patterns in regulation and five genes (*Agt*, *E2f6*, *Kcnj8*, *Me1* and *Pdk1*) exhibited more complex trends that appeared to vary with tissues. Two genes in particular (*Enpp1* and *Pdk1*) possessed both AHRE-I (Full) and AHRE-II binding motifs in the upstream 5′-regulatory region while four other genes (*Car3*, *Ctsc*, *Gatm* and *Kcnj8*) possessed only the AHRE-II motif. Although *Gstm4* and *Slc35a3* were not found to contain either motif, both showed evidence of AHR binding *in vitro* [57].

### Constitutive AHR pathways involve many common genes

**Figure 4:**
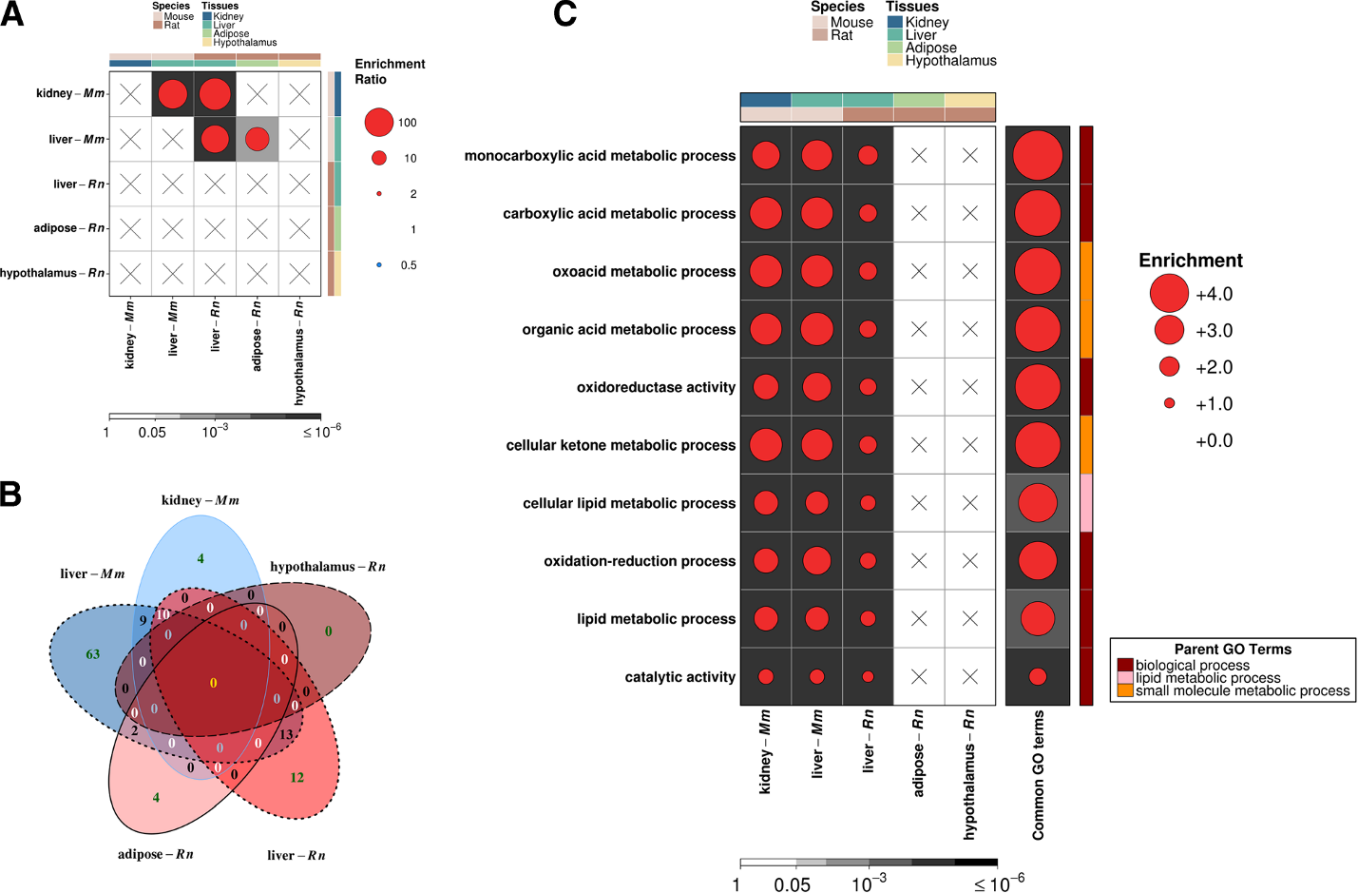
**Assessment of pathway similarity.** Hypergeometric testing was conducted on all pairs of tissues to identify commonly enriched biological pathways **(A)**. Magnitude of enrichment is represented by spot size while background shade represents *q*-values from hypergeometric testing. “X” denotes absence of significant enrichment. Assessment of common GO terms across all species and tissues revealed localized overlap **(B)**. Three general ontologies, biological process, lipid metabolic process and molecular function, are represented among the ten commonly enriched pathways **(C)**.

To assess enrichment of specific pathways in biological interactions, we conducted gene ontology (GO) analysis using GoMiner [64]. Our analysis did not unveil any enriched pathways in the rat hypothalamus data but did identify several significantly enriched ontology terms amongst the other tissues. To capture common pathways, we analyzed overlapping GO terms across tissues and species and conducted hypergeometric testing to assess the significance of overlapping pathways. Raw counts of GO term overlap can be found in Additional file 27: Figure S16. We observed that the enriched ontologies in rat adipose and mouse kidney were entirely independent of one another (Figure 4A). However for most other tissues we found significant overlap, ranging from 50 – 180 times greater than the expected count. While the overlap of mouse kidney and mouse liver at the gene level was enriched by 3.7 fold (Figure 2A), at the pathway level it was nearly 110 fold (observed = 19, expected = 0.17, *q* = 2.75 × 10^-37^, Figure 4A). Similarly between mouse and rat liver, there were 93 times more GO terms in common than expected by chance alone (observed = 25, expected = 0.27, *q* = 1.91 × 10^-45^), in contrast to 2.3 times the number of shared genes (Figure 2A). Pairwise assessments of all GO term overlaps are available in Additional file 28: Figure S17.Results at the pathway level further verified variations at the transcriptomic level as a function of species and tissues, but also demonstrated an abundance of biological interactions within excretory tissues, such as kidney and liver, relative to the adipose or hypothalamus (Figure 4B). We selected genes that were significant in at least three tissues and re-analyzed functional enrichment using GoMiner to assess global trends. We filtered our new results for GO terms that were also reflected in at least three of the individual analyses. We found ten such commonly enriched GO terms and traced back their branch of functional groupings to identify the originating parent GO terms (Figure 4C). We found that all ten can be essentially explained by three unique parent ontologies of (fundamental) biological processes, lipid metabolic processes and small molecule metabolic processes.

### AHR constitutive transcriptomic effects regulated primarily by direct binding

**Figure 5:**
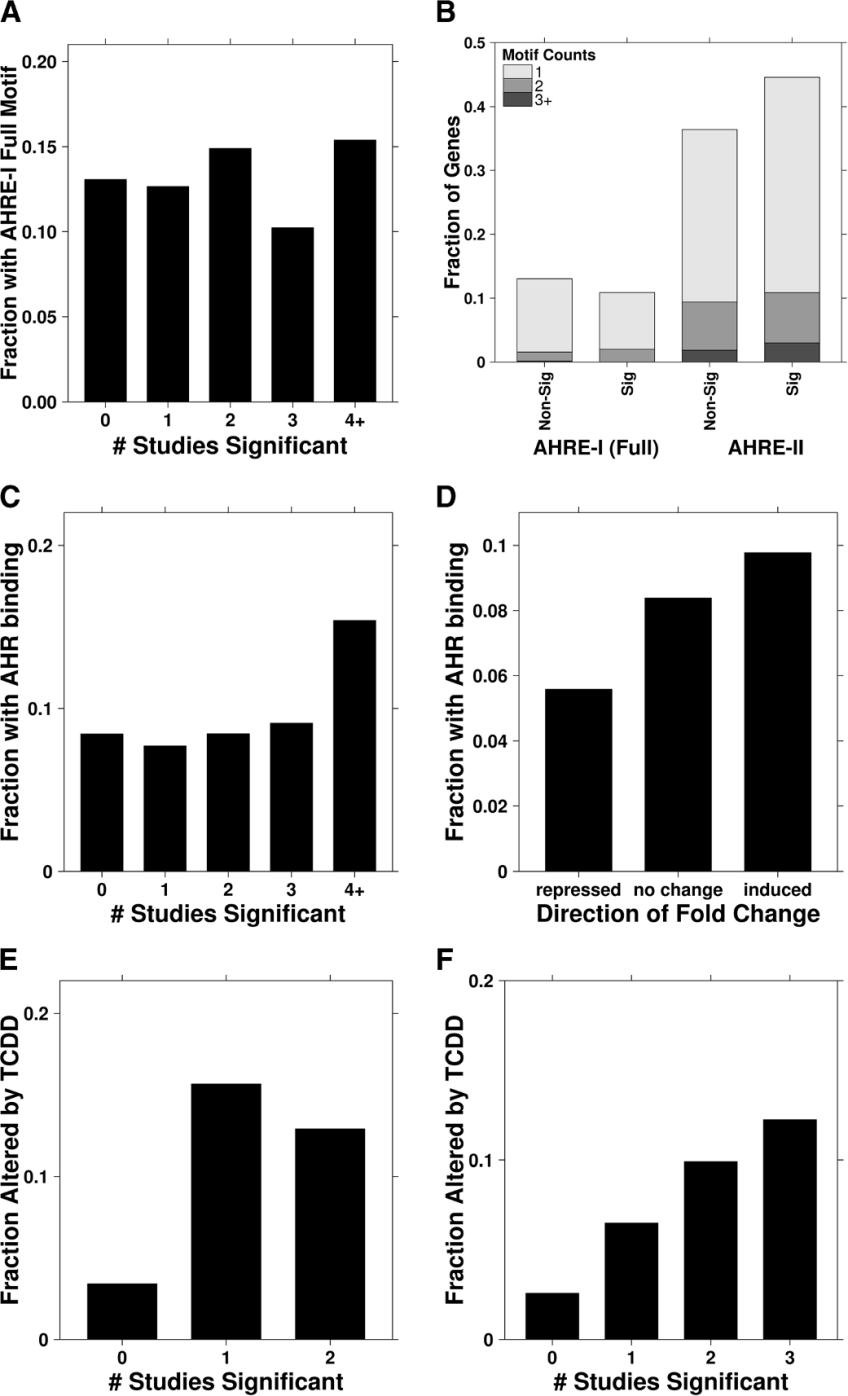
**Evaluation of AHR binding: presence and effects.** A higher fraction of genes that were significantly affected by AHR-status across multiple tissues were found to possess AHRE-I (Full) motifs in the upstream 5′-regulatory region, compared to genes that were AHR-status independent **(A)**. At several count thresholds, the fractions of significant and non-significant genes were contrasted for the presence of both AHRE-I (Full) and AHRE-II motifs **(B)**. Similar to findings of the transcription factor binding site analysis, a higher fraction of genes significantly altered across multiple tissues were found to exhibit AHR binding *in vitro* **(C)**. The AHR appears to largely exert an upregulating effect on genes in rat liver in the absence of exogenous ligands, but a smaller fraction of downregulation is present as well **(D)**. Genes that were differentially-abundant in the constitutive condition were also observed to be altered in greater proportions following TCDD-induced AHR activation in the mouse liver **(E)** and rat liver **(F)**.

Lastly, we sought to determine whether the changes we observed at the constitutive level in animals of different AHR-status were mediated directly by the AHR. We took three separate approaches to address this question. First, we examined the presence of conserved AHR binding motifs in the upstream 5′-regulatory regions of significantly altered genes to assess potential for AHR regulation resulting from direct binding. We found that the presence of conserved AHRE-I (Full) motifs was generally more common amongst genes whose abundance varied with AHR-status in multiple tissues (Figure 5A). Corresponding fractions for the AHRE-I (Core), AHRE-I (Extended) and AHRE-II motifs are available in Additional file 29: Figure S18. Next, we conducted a more inclusive assessment and considered the partitions of these genes for both AHRE-I (Full) and AHRE-II motifs. To facilitate this analysis, we dichotomized our gene list by redefining “significant” and “non-significant” with respect to an alternate selection criterion: differential abundance in at least three tissues. We calculated the fractions with one, two or ≥ three motif counts and found that in general, “significant” genes contained more occurrences of AHRE-II motifs (Figure 5B).

Our second approach was to assess AHR binding using a publicly available ChIP-chip dataset [57]. Mirroring the procedure of our first approach, we evaluated the fraction of genes to exhibit AHR binding in the absence of exogenous ligands (*p* < 0.05). We found a higher fraction of binding amongst genes commonly altered across multiple tissues (Figure 5C). Focusing on the rat liver data, which displayed higher magnitude changes in the proposed AHR constitutive genes (Figure 3B), we split the transcriptome into three groups based on their direction of regulation: significantly-repressed genes, significantly up-regulated genes and genes with no statistically-significant changes. We found that induced genes displayed more binding (Figure 5D), suggesting that AHR binding generally elicits an activating effect.

Our final approach was to assess whether those genes that differ in constitutive expression also differ in response to TCDD. For this, we compared our findings to genes that were significantly altered in TCDD-treated samples from our mouse and rat liver experiments [37, 46]. We found greater proportions of genes that were changed by TCDD treatment in the mouse liver (Figure 5E) and rat liver (Figure 5F) to also exhibit significant variations in their constitutive expressions between AHR-variant animals. In contrast, genes that remained unresponsive to TCDD were also largely invariant with AHR status (Figures 5E,5F). Since TCDD is a known activator of the AHR, seeing similar trends in the presence and absence of exogenous ligands provides strong support of regulatory control by the AHR of many genes that also respond to dioxin-like chemicals.

## Discussion

The AHR is highly conserved throughout evolution; this fact alone suggests that it plays an important role in organism development and function [65]. We previously reported hints that gene expression in control rat livers depend on AHR-status, but this was limited to a single tissue and species [36]. Here, we generalize and extend that result by evaluating constitutive mRNA associations with AHR activity in two species and four tissues. Although we refer to H/W rats throughout the text as “AHR-less-active” animals, we are aware of the bold premise in assuming lower AHR activity in these animals, especially since *Cyp1a1* induction by TCDD occurs normally in the H/W strain [37]. However, we do generally observe lower numbers of genes altered as a result of TCDD treatment in comparison to L-E rats [37], suggesting that inter-strain differences in AHR physiology are present [37] and that our assumption is not unfounded. Evidence in support of our approach is further observed in results from the rat liver samples, where mRNA abundance of the *Ahr* was significantly lower for H/W rats (Figure 3A). Thus we decided to maintain the usage of this terminology throughout. After rigorous statistical analysis and attempts to control for species-specific effects, we identified 13 genes of interest: *Agt*, *Car3*, *Creg1*, *Ctsc*, *E2f6*, *Enpp1*, *Gatm*, *Gstm4*, *Kcnj8*, *Me1*, *Pdk1*, *Slc35a3*, and *Sqrdl* (Figure 3B). We propose that these genes are likely AHR-regulated via endogenous ligands in multiple tissues and species.

Three genes have roles in energy homeostasis: *Me1*, *Pdk1* and *Gatm. Me1* encodes malic enzyme 1, a lipogenic enzyme essential for catalyzing the production of pyruvate from malate in the citric acid cycle for fatty acid synthesis [66]. In recent years, it has been identified as a causal gene in the development of obesity and type II diabetes from genome-wide association studies [66–68]. Pyruvate serves as an intermediary between glucose, fatty acid and amino acid metabolic pathways and is an important molecule in the regulation of body energy. We postulate that the lower hepatic levels of *Me1* observed in AHR-less-active animals may suggest a lower propensity for lipogenesis in these animals and a preferred reliance on an alternative pathway for energy catabolism. This may be a pathway not directly influenced by the AHR, which could explain the increased resistance of these animals to TCDD-induced wasting syndrome [61]. Interestingly, the log_2_-fold change of *Me1* following TCDD-treatment in H/W rat liver was 2.2 (*q* = 0.02) but nearly twice as high in L-E rats (log_2_-fold change = 4.24, *q* = 0.04) [37].

*Pdk1* is also involved in the regulation of pyruvate [69]. It encodes isozyme 1 of the pyruvate dehydrogenase kinase, a mitochondrial enzyme responsible for the phosphorylation and subsequent inactivation of pyruvate dehydrogenase [69]. Since pyruvate dehydrogenase functions to increase acetyl-CoA production, *Pdk1* thereby exerts an inhibitory effect on lipogenesis. Lower hepatic levels of *Pdk1* observed in AHR-less-active animals are consistent with the previously-stated hypothesis of decreased lipogenesis in AHR-less-active animals. The third gene, *Gatm*, encodes glycine amidinotransferase, an enzyme whose net effect is to increase production of creatine. Creatine is a molecule that supplies energy to muscle cells via increasing ATP formation [70]. mRNA levels of *Gatm* are higher in AHR-less-active animals, indicating that on average, these animals utilize creatine more than AHR-active animals as a source of energy. Taken in conjunction with levels of *Me1* and *Pdk1*, it appears that AHR-less-active animals may preferentially utilize nitrogenous molecules as sources of energy and that this preference may somehow confer a protective effect against TCDD-induced wasting syndrome.

Several other genes, though not functionally categorized together, have important implications on the developmental role of the AHR. Two of these have roles in vascularization: *Agt* and *Kcnj8. Agt*, which encodes angiotensinogen, is important for the regulation of blood pressure [71]. Mutations in this gene have been associated with renal tubular dysgenesis [72, 73]. This is particularly relevant as a known teratogenic effect of TCDD is hydronephrosis; thus dysregulation of *Agt* may be contributing to this outcome [74]. Moreover, studies in AHR knockout mice have demonstrated reduced renin-angiotensin signalling as a cause of hypotension in these animals [75]. *Kcnj8* encodes a G-protein controlled potassium channel that preferentially directs potassium into the cell and is critical for vascular tonus [76]. Knockout mice for *Kcnj8* demonstrate high rates of spontaneous death resulting from cardiovascular events [76]. Since cardiomyopathy and vascular hypertrophy are both documented phenotypes of AHR-null mice, a rational biological backdrop for AHR involvement in their regulation exists.

*Enpp1* encodes the enzyme ectonucleotide pyrophosphatase/phosphodiesterase-1, which is important for the inhibition of calcification and maintenance of insulin sensitivity [77]. *Enpp2* was also one of the relatively few H/W-specific genes that were altered by TCDD in rat hepatic tissues [37]. Previous studies have shown that the AHR is required both for proper vessel pruning and insulin regulation, which again suggests that AHR physiological control of this gene is coherent with existing biological evidence [78, 79]. *Creg1*, cellular repressor of E1A-stimulated genes 1, encodes a protein that antagonizes the activity of the adenovirus E1A protein, which functions to increase proliferation and decrease differentiation [80, 81]. Higher mRNA levels of this proliferation-antagonizing gene were found in AHR-less-active animals, suggesting that chronic AHR activation may lead to higher levels of the proliferative adenovirus E1A protein. This implicates a role for AHR in carcinogenesis, an aspect of AHR physiology that has been studied quite extensively [13, 20, 31, 82–91].

Our previous studies proved gene ontology analysis to be a useful tool in comparing and grouping significantly altered genes for a more comprehensive assessment of biological relevance [35, 44]. For our current analysis, we noted a greater overlap at the pathway level compared to the gene level and this was strongly indicative of the common recruitment of other genes amongst these pathways and processes, in a manner that cannot be accurately assessed based on transcriptomic changes alone. We found a major fraction of genes influenced by AHR-status to be associated with lipid metabolism processes (Figure 4C). This observation was also made in our previous studies of TCDD-induced transcriptomic variation [61, 92, 93]. Enrichment of changed genes involved in lipid metabolic processes is consistent with our previous proposal of inherent differences in energy regulation between AHR-variant animals and may pose as a potential explanation for differential sensitivity to TCDD-induced body wasting.

Our AHR binding analysis provided strong indications of AHR binding for AHR-related differences in the constitutive regulation of genes. We found a high fraction of genes whose constitutive expression depends on AHR-status to contain AHR-DNA binding motifs as well as exhibiting AHR binding *in vitro* (Figure 5A,5C). However, the transcriptomic effects of AHR binding for the constitutive targets may be down-regulatory, as higher levels of these genes were observed in the AHR-less-active animals (Figure 3B). We postulate this may be due to the presence of inhibitory dioxin-response elements (iDREs) [94]. Binding of the activated AHR complex to specific DNA sequences, termed inhibitory dioxin-response elements (iDREs), exerts anti-estrogenic effects [94]. These iDREs either overlap or are immediately adjacent to response elements, so binding of the activated AHR complex blocks binding of other transcription factors, preventing transcription [94–96]. The removal of this inhibition in our AHR-less-active animals may have resulted in the higher mRNA abundances observed for certain genes (Figure 3B). Finally, since binding is not an absolute necessity for regulation, we speculate that genes without evidence of binding sites may be regulated via other means, such as interactions of the AHR complex with other transcription factors [97].

There are still some issues concerning cross-species comparisons and caution must be taken when drawing conclusions from these results. As indicated in Figure 2C, upwards of 1000 genes were altered as a result of AHR-status in rat liver and rat hypothalamus, but less than 300 were affected in the mouse tissues. Theoretically, the rat model, which captures the effects of a deletion in the transactivation domain of the AHR, should not have more profound effects than the mouse model, which employed a complete knockout of the receptor. One potential explanation might be the molecular context of these assessments: the mouse model measured the effects of the AHR within the same strain (C57BL/6J mice) while the rat model actually compared differences across strains (H/W *vs.* L-E rats). The genomes of different strains of rats will likely vary at many loci, whereas in our mouse model, this difference was virtually – if not exclusively – confined to the AHR locus. A portion of the detected changes in rats may be artefacts of strain variations. A better cross-species parallel might utilize the DBA/2 mouse, with the AHR allele of reduced binding affinity, in the murine model [98]. Unfortunately, we do not possess array data for mice with these alleles and our current cross-species-and-tissues analysis was done using the best available data.

A similar but distinct issue exists for our rat model. As previously mentioned, both H/W and L-E rats exhibit comparable hepatic induction of *Cyp1a1* upon treatment with TCDD [37]. This may be suggestive of inter-strain differences in the AHR pathways, which bears restrictions on the accurate interpretation and generalizability of the results. Alternatively, the use of knockout rat models would greatly improve our comparison and mitigate inter-strain uncertainties. Unfortunately, knockout rats were not technically feasible until recently and presently only a few specific models have been made commercially available [99]. We anticipate that advancing technology will aid in both increasing the availability and lowering the costs of using such models and enable us to further explore this question in the future.

It is important to acknowledge that although animals used in these studies were not treated with exogenous ligands per se, all were given a single dose of corn-oil. Responses to corn oil cannot be ruled out for our studies using rats. However, this is not the case for our mouse experiments, where AHR activity has been effectively knocked-out. Therefore the effects we do observe are via AHR-mediation, even if triggered to some extent by corn oil. Finally, it is important to note that our tests were conducted across all genes and not only amongst expressed genes. In other words, for our cross-tissues comparisons, we did not filter out genes that were only expressed in some tissues (*i.e.* liver-specific, kidney-specific or other tissue-specific genes). By using the total number of genes as the denominator, we may be underestimating the fraction of common effects across tissues.

## Conclusions

In summary, we propose a list of 13 genes that constitute a constitutive AHR gene battery, at least in the tissues studied, in mice and rats (*Agt, Car3, Creg1, Ctsc, E2f6, Enpp1, Gatm, Gstm4, Kcnj8, Me1, Pdk1, Slc35a3, and Sqrdl*)*.* We established these candidates from within-experiment transcriptional profiling of control animals with distinct AHR genotypes, using appropriate statistical methods and assessed their mode of regulation via AHRE and AHR binding analyses. We propose that the majority of these genes are inversely associated with AHR activity and although binding is the primary method of transcriptional regulation, some of these genes could also be regulated via non-classic mechanisms, such as interactions with other transcription factors. We further conducted gene ontology analysis to make large-scale interpretations and to suggest possible functional connections with existing knowledge of AHR function. We hypothesize that AHR-less-active animals differ on a fundamental molecular level from AHR-active animals in how energy balance is regulated. Future work will help determine whether the genes identified in this study, as being dependent on AHR-status for constitutive expression, are exclusive to mice and rats, or whether they are also functional in other species and cell types. We hope that this work will rally further research into an often under-appreciated aspect of AHR physiology.

### Availability of supporting data

The data sets supporting the results of this article are available in the National Center for Biotechnology Information (NCBI) Gene Expression Omnibus (GEO) repository, through accession numbers GSE15857 for mouse kidney (http://www.ncbi.nlm.nih.gov/geo/query/acc.cgi?acc=GSE15857), GSE15858 for mouse liver (http://www.ncbi.nlm.nih.gov/geo/query/acc.cgi?acc=GSE15858), GSE18301 for rat adipose (http://www.ncbi.nlm.nih.gov/geo/query/acc.cgi?acc=GSE18301), GSE18257 for rat hypothalamus (http://www.ncbi.nlm.nih.gov/geo/query/acc.cgi?acc=GSE18257) and GSE13513 for rat liver (http://www.ncbi.nlm.nih.gov/geo/query/acc.cgi?acc=GSE13513).

## Electronic supplementary material

Additional file 1: Figure S1: Data Quality Assessment: Mouse, Kidney. Comparison of distributions of probe-level log_2_ intensities before (A) and after (B) RMA normalization. The average intensities of probes across ProbeSets were examined using an RNA degradation plot (C). Inter-array correlation was assessed with a heatmap generated using complete agglomerative clustering, with Pearson’s coefficient employed as the similarity metric (D). (PDF 411 KB)

Additional file 2: Figure S2: Data Quality Assessment: Mouse, Liver. Comparison of distributions of probe-level log_2_ intensities before (A) and after (B) RMA normalization. The average intensities of probes across ProbeSets were examined using an RNA degradation plot (C). Inter-array correlation was assessed with a heatmap generated using complete agglomerative clustering, with Pearson’s coefficient employed as the similarity metric (D). (PDF 392 KB)

Additional file 3: Figure S3: Data Quality Assessment: Rat, Liver. Comparison of distributions of probe-level log_2_ intensities before (A) and after (B) RMA normalization. The average intensities of probes across ProbeSets were examined using an RNA degradation plot (C). Inter-array correlation was assessed with a heatmap generated using complete agglomerative clustering, with Pearson’s coefficient employed as the similarity metric (D). (PDF 408 KB)

Additional file 4: Figure S4: Data Quality Assessment: Rat, Adipose. Comparison of distributions of probe-level log_2_ intensities before (A) and after (B) RMA normalization. The average intensities of probes across ProbeSets were examined using an RNA degradation plot (C). Inter-array correlation was assessed with a heatmap generated using complete agglomerative clustering, with Pearson’s coefficient employed as the similarity metric (D). Presence of an outlier array was evident in the L-E group (RAE2302_083106W_AO07.CEL). (PDF 430 KB)

Additional file 5: Figure S5: Data Quality Assessment: Rat, Hypothalamus. Comparison of distributions of probe-level log_2_ intensities before (A) and after (B) RMA normalization. The average intensities of probes across ProbeSets were examined using an RNA degradation plot (C). Intra-array correlation was assessed with a heatmap generated using complete agglomerative clustering, with Pearson’s coefficient employed as the similarity metric (D). (PDF 403 KB)

Additional file 6: Figure S6: Data Quality Assessment: Rat, Adipose, Outlier Removed. Comparison of distributions of probe-level log_2_ intensities before (A) and after (B) RMA normalization with the outlier array removed (RAE2302_083106W_AO07.CEL). The average intensities of probes across ProbeSets were examined using an RNA degradation plot (C). Intra-array correlation was assessed with a heatmap generated using complete agglomerative clustering, with Pearson’s coefficient employed as the similarity metric (D). Removal of the outlier array improved overall spatial and distributional homogeneity. (PDF 400 KB)

Additional file 7: Table S1: Annotated Genes and Fold Changes: Mouse, Kidney. The log_2_ fold changes (M) and *q*-values of all 17,607 unique and sorted Entrez Gene IDs (GeneID) are listed. Useful gene information are provided where available, including gene symbol (Symbol), chromosome, HomoloGene ID (HID) and gene full name (FullName). (TXT 1 MB)

Additional file 8: Table S2: Annotated Genes and Fold Changes: Mouse, Liver. The log_2_ fold changes (M) and *q*-values of all 17,607 unique and sorted Entrez Gene IDs (GeneID) are listed. Useful gene information are provided where available, including gene symbol (Symbol), chromosome, HomoloGene ID (HID) and gene full name (FullName). (TXT 1 MB)

Additional file 9: Table S3: Annotated Genes and Fold Changes: Rat, Liver. The log_2_ fold changes (M) and *q*-values of all 12,560 unique and sorted Entrez Gene IDs (GeneID) are listed. Useful gene information are provided where available, including gene symbol (Symbol), chromosome, HomoloGene ID (HID) and gene full name (FullName). (TXT 1 MB)

Additional file 10: Table S4: Annotated Genes and Fold Changes: Rat, Adipose. The log_2_ fold changes (M) and *q*-values of all 12,560 unique and sorted Entrez Gene IDs (GeneID) are listed. Useful gene information are provided where available, including gene symbol (Symbol), chromosome, HomoloGene ID (HID) and gene full name (FullName). (TXT 1 MB)

Additional file 11: Table S5: Annotated Genes and Fold Changes: Rat, Hypothalamus. The log_2_ fold changes (M) and *q*-values of all 12,560 unique and sorted Entrez Gene IDs (GeneID) are listed. Useful gene information are provided where available, including gene symbol (Symbol), chromosome, HomoloGene ID (HID) and gene full name (FullName). (TXT 1 MB)

Additional file 12: Table S6: Chromosome Enrichment. Hypergeometric testing was used to assess chromosomal bias of significant genes. The table reports the observed counts, expected counts and adjusted *p*-values of the test. (XLS 12 KB)

Additional file 13: **R Code for Generating Bootstrap P-value.** The R code used to generate the bootstrap *p*-value for the overlap between three rat tissues. (PDF 24 KB)

Additional file 14: Figure S7: Differential Power Analysis: Rat, Adipose. To assess the effect of removing one L-E outlier (RAE2302_083106W_AO07.CEL) on statistical power, one array from the H/W group was systematically removed and the data re-normalized and re-fitted. Similar patterns of *q*-value densities were observed following each removal, justifying proceeding with subsequent analyses using all H/W arrays. (PDF 1 MB)

Additional file 15: Figure S8: Transcription Factor Binding Analysis: Rats, Motif Counts. Kernel densities of counts for AHRE-I (Core), AHRE-I (Extended), AHRE-I (Full) and AHRE-II motifs are shown for significant (A) and non-significant (B) genes in rat tissues. The median is represented by the circular point while the 90th percentile is represented by the diamond point. (PDF 953 KB)

Additional file 16: Figure S9: Transcription Factor Binding Analysis: Mice, Motif Counts. Kernel densities of counts for AHRE-I (Core), AHRE-I (Extended), AHRE-I (Full) and AHRE-II motifs are shown for significant (A) and non-significant (B) genes in mouse tissues. The median is represented by the circular point while the 90th percentile is represented by the diamond point. (PDF 638 KB)

Additional file 17: Figure S10: Transcription Factor Binding Analysis: Rats, Motif Scores. Kernel densities of scores for AHRE-I (Core), AHRE-I (Extended), AHRE-I (Full) and AHRE-II motifs are shown for significant (A) and non-significant (B) genes in rat tissues. The median is represented by the circular point while the 90th percentile is represented by the diamond point. (PDF 949 KB)

Additional file 18: Figure S11: Transcription Factor Binding Analysis: Mice, Motif Scores. Kernel densities of scores for AHRE-I (Core), AHRE-I (Extended), AHRE-I (Full) and AHRE-II motifs are shown for significant (A) and non-significant (B) genes in mouse tissues. The median is represented by the circular point while the 90th percentile is represented by the diamond point. (PDF 656 KB)

Additional file 19: Table S7: GO Analysis: Mouse, Kidney. A list of significantly enriched GO terms (FDR < 0.01) with reported enrichment, statistical significance, GO term definition, list of changed genes and parent GO term information. (TXT 5 KB)

Additional file 20: Table S8: GO Analysis: Mouse, Liver. A list of significantly enriched GO terms (FDR < 0.01) with reported enrichment, statistical significance, GO term definition, list of changed genes and parent GO term information. (TXT 27 KB)

Additional file 21: Table S9: GO Analysis: Rat, Adipose. A list of significantly enriched GO terms (FDR < 0.01) with reported enrichment, statistical significance, GO term definition, list of changed genes and parent GO term information. (TXT 813 bytes)

Additional file 22: Table S10: GO Analysis: Rat, Liver. A list of significantly enriched GO terms (FDR < 0.01) with reported enrichment, statistical significance, GO term definition, list of changed genes and parent GO term information. (TXT 26 KB)

Additional file 23: Figure S12: *p*-Value Sensitivity: count. The counts of significant genes after linear fitting and multiple testing correction were determined to be threshold-independent based on *p*-value sensitivity analysis. (PDF 1 MB)

Additional file 24: Figure S13: *p*-Value Sensitivity: direction. *p*-value sensitivity analysis revealed that the results were equally sensitive for detection of up- and down-regulated genes. (PDF 1 MB)

Additional file 25: Figure S14: Raw Counts of Overlapping Genes. The counts of genes common to two tissues are shown for every tissue pair, with the magnitude of overlap represented by spot size and background shade denoting *q*-values calculated from hypergeometric testing. (PDF 655 KB)

Additional file 26: Figure S15: Gene Overlap Between Datasets. Overlapping genes are shown for mouse liver *vs.* mouse kidney (A), mouse liver *vs.* rat hypothalamus (B), mouse kidney *vs.* rat adipose (C), rat adipose *vs.* rat hypothalamus (D), mouse kidney *vs.* rat hypothalamus (E), rat adipose *vs.* rat liver (F), mouse kidney *vs.* rat liver (G), rat liver *vs.* rat hypothalamus (H) and mouse liver *vs.* rat adipose (I). Blue and red represent mouse and rat tissues respectively. Dotted, solid, dashed and no lines are used to visually differentiate liver, adipose, hypothalamus and kidney tissues. (PDF 639 KB)

Additional file 27: Figure S16: Raw Counts of Overlapping GO terms. The counts of GO terms common to two tissues are shown for every tissue pair, with the magnitude of overlap represented by spot size and background shade denoting *q*-values calculated from hypergeometric testing. (PDF 622 KB)

Additional file 28: Figure S17: GO Term Overlap Between Datasets. Overlapping GO terms are shown for mouse liver *vs.* mouse kidney (A), mouse kidney *vs.* rat liver (B), mouse liver *vs.* rat adipose (C), mouse kidney *vs.* rat adipose (D), mouse kidney *vs.* rat hypothalamus (E), mouse liver *vs.* rat hypothalamus (F), mouse liver *vs.* rat liver (G), rat adipose *vs.* rat hypothalamus (H), rat liver *vs.* rat adipose (I) and rat liver *vs.* rat hypothalamus (J). Blue and red represent mouse and rat tissues respectively. Dotted, solid, dashed and no lines are used to visually differentiate liver, adipose, hypothalamus and kidney tissues. (PDF 646 KB)

Additional file 29: Figure S18: Fraction of genes with TFBS motifs. A comparison of genes differentially-abundant across multiple tissues and their fractions of observed AHRE-I (Core) (A), AHRE-I (Extended) (B) and AHRE-II (C) motifs. (PDF 876 KB)
