# Supplementary figures and images for: Cross-species transcriptomic analysis elucidates constitutive aryl hydrocarbon receptor activity
Source: BMC Genomics. 2014 Dec 3;15(1):1053. doi: 10.1186/1471-2164-15-1053 (PMC4301818; doi:10.1186/1471-2164-15-1053)

# Data Quality Assessment

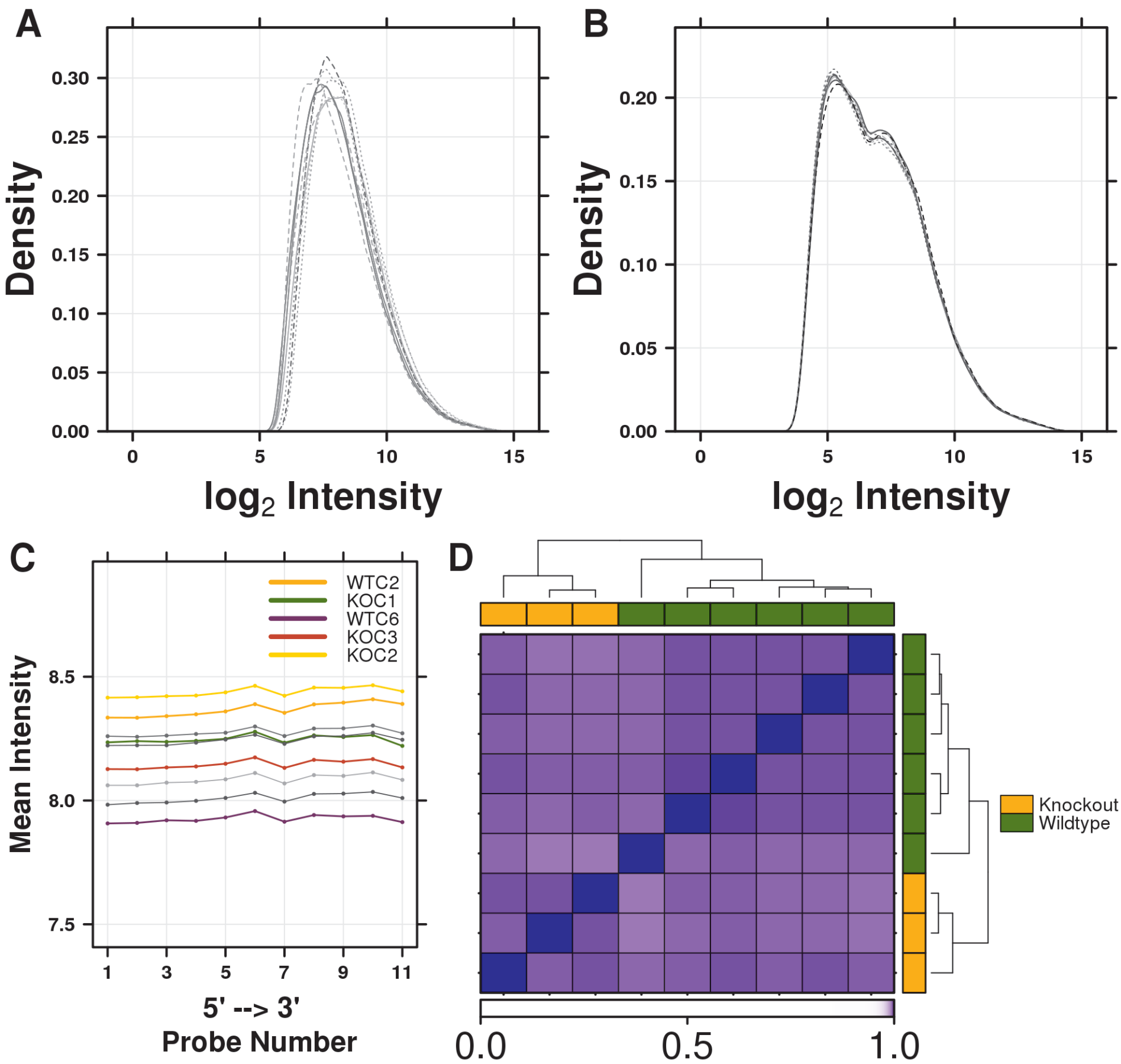

Supplement: Supplementary file 1 — Additional file 1: Figure S1: Data Quality Assessment: Mouse, Kidney. Comparison of distributions of probe-level log2 intensities before (A) and after (B) RMA normalization. The average intensities of probes across ProbeSets were examined using an RNA degradation plot (C). Inter-array correlation was assessed with a heatmap generated using complete agglomerative clustering, with Pearson’s coefficient employed as the similarity metric (D). (PDF 411 KB) [file 12864_2014_6766_MOESM1_ESM.pdf]

# Data Quality Assessment

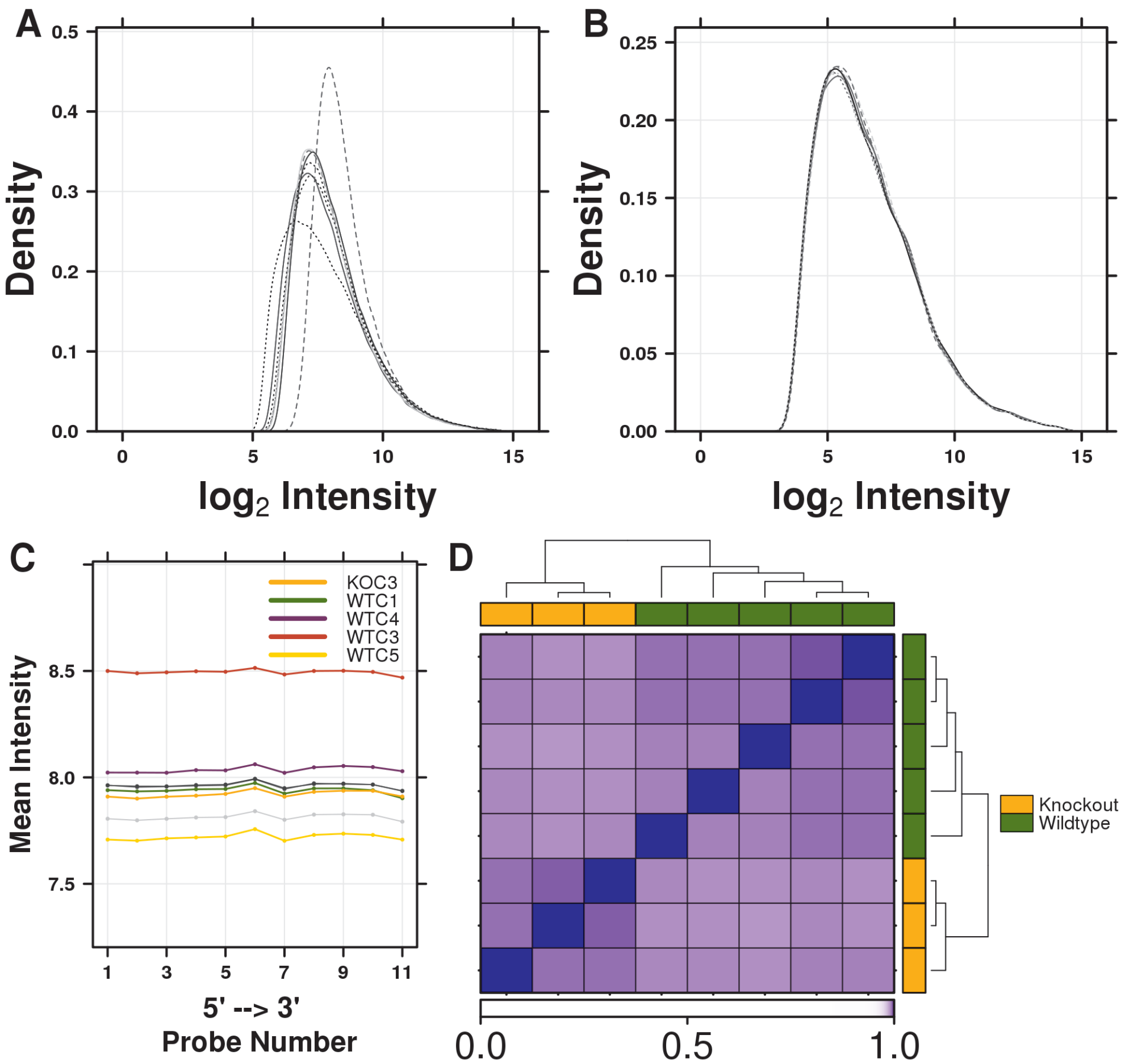

Supplement: Supplementary file 2 — Additional file 2: Figure S2: Data Quality Assessment: Mouse, Liver. Comparison of distributions of probe-level log2 intensities before (A) and after (B) RMA normalization. The average intensities of probes across ProbeSets were examined using an RNA degradation plot (C). Inter-array correlation was assessed with a heatmap generated using complete agglomerative clustering, with Pearson’s coefficient employed as the similarity metric (D). (PDF 392 KB) [file 12864_2014_6766_MOESM2_ESM.pdf]

# Data Quality Assessment

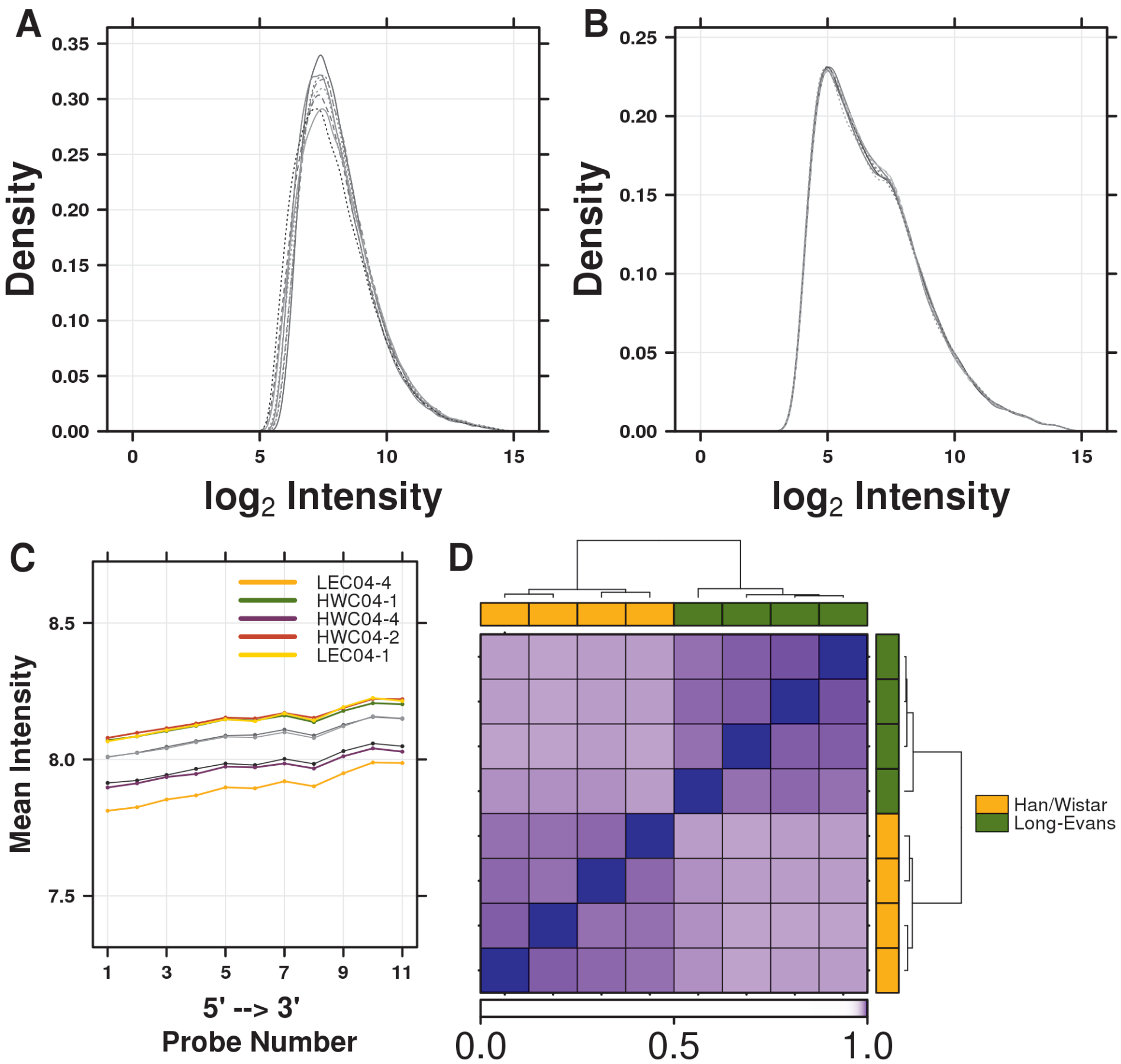

Supplement: Supplementary file 3 — Additional file 3: Figure S3: Data Quality Assessment: Rat, Liver. Comparison of distributions of probe-level log2 intensities before (A) and after (B) RMA normalization. The average intensities of probes across ProbeSets were examined using an RNA degradation plot (C). Inter-array correlation was assessed with a heatmap generated using complete agglomerative clustering, with Pearson’s coefficient employed as the similarity metric (D). (PDF 408 KB) [file 12864_2014_6766_MOESM3_ESM.pdf]

# Data Quality Assessment

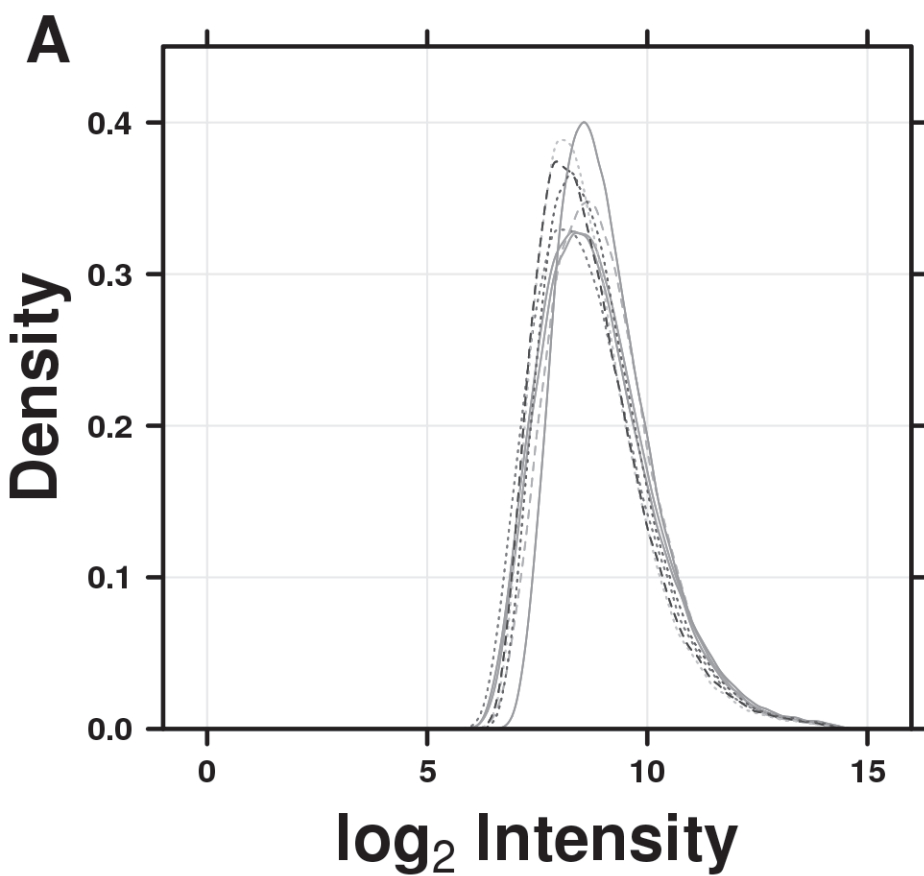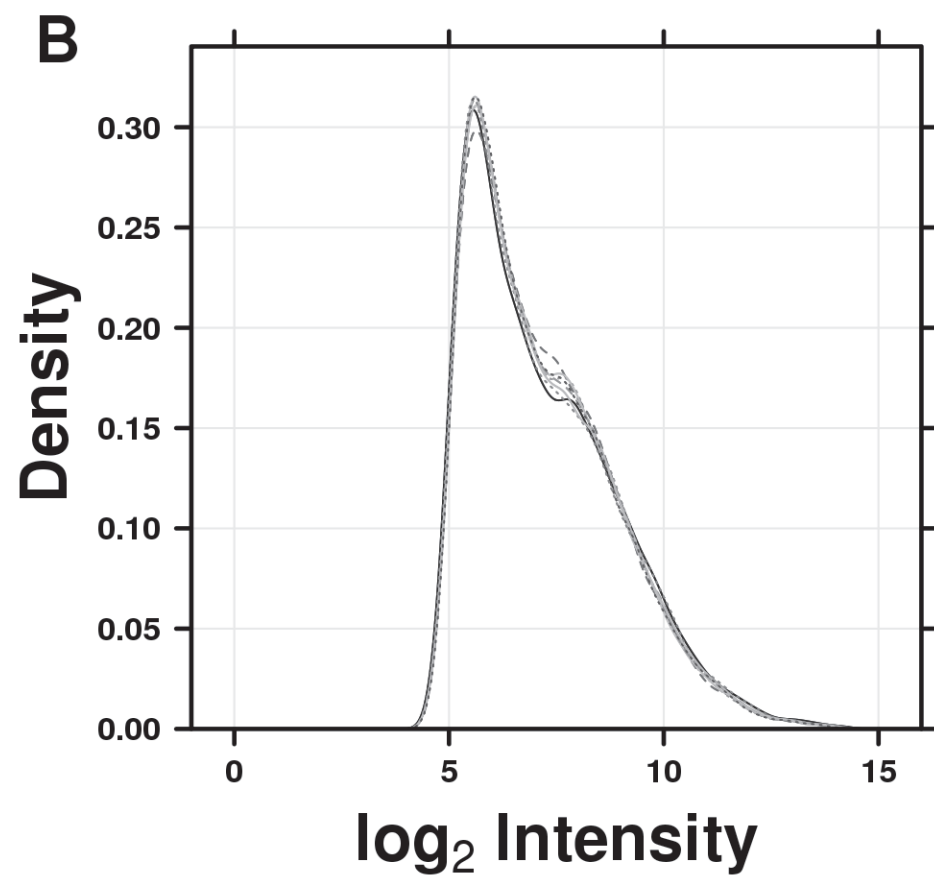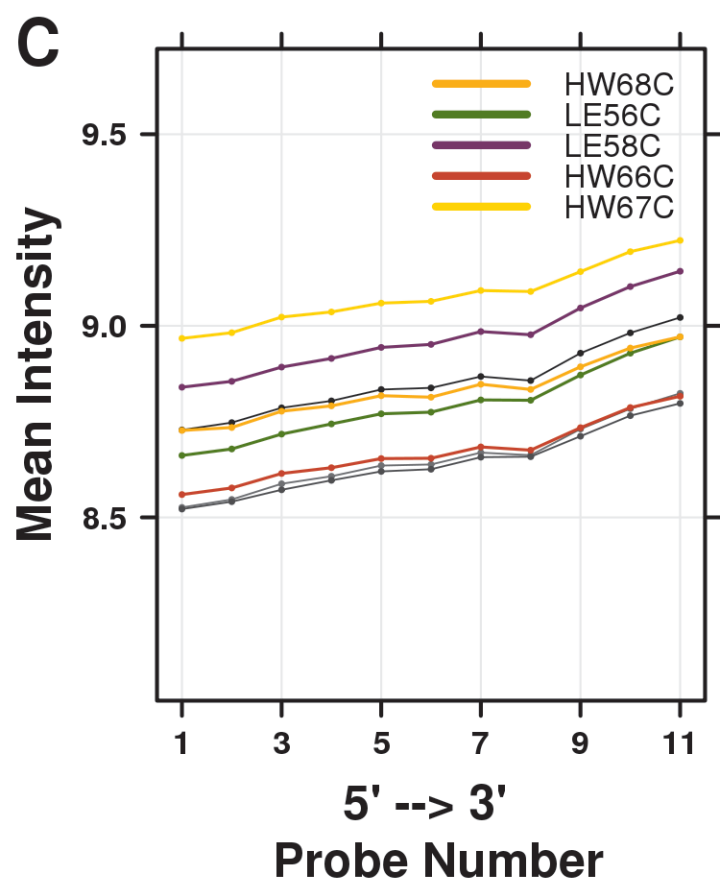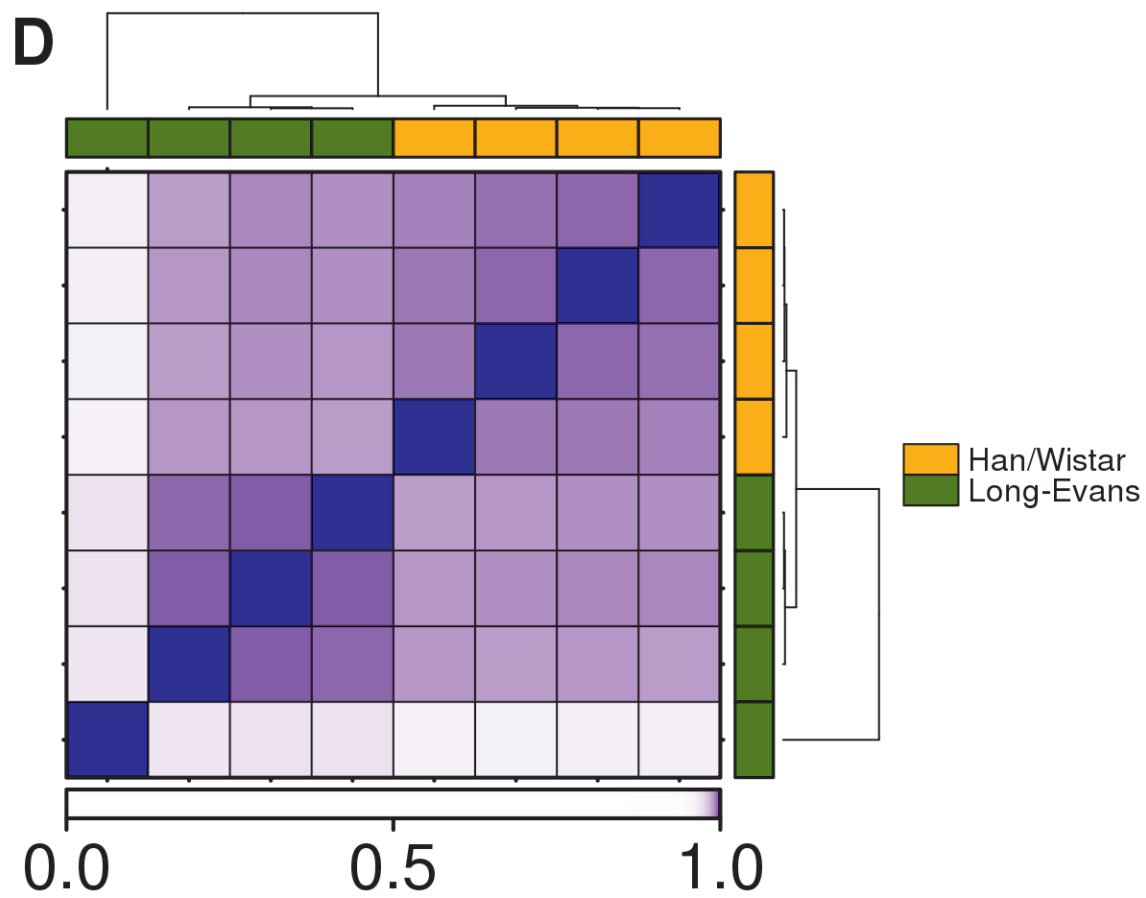

Supplement: Supplementary file 4 — Additional file 4: Figure S4: Data Quality Assessment: Rat, Adipose. Comparison of distributions of probe-level log2 intensities before (A) and after (B) RMA normalization. The average intensities of probes across ProbeSets were examined using an RNA degradation plot (C). Inter-array correlation was assessed with a heatmap generated using complete agglomerative clustering, with Pearson’s coefficient employed as the similarity metric (D). Presence of an outlier array was evident in the L-E group (RAE2302_083106W_AO07.CEL). (PDF 430 KB) [file 12864_2014_6766_MOESM4_ESM.pdf]

# Data Quality Assessment

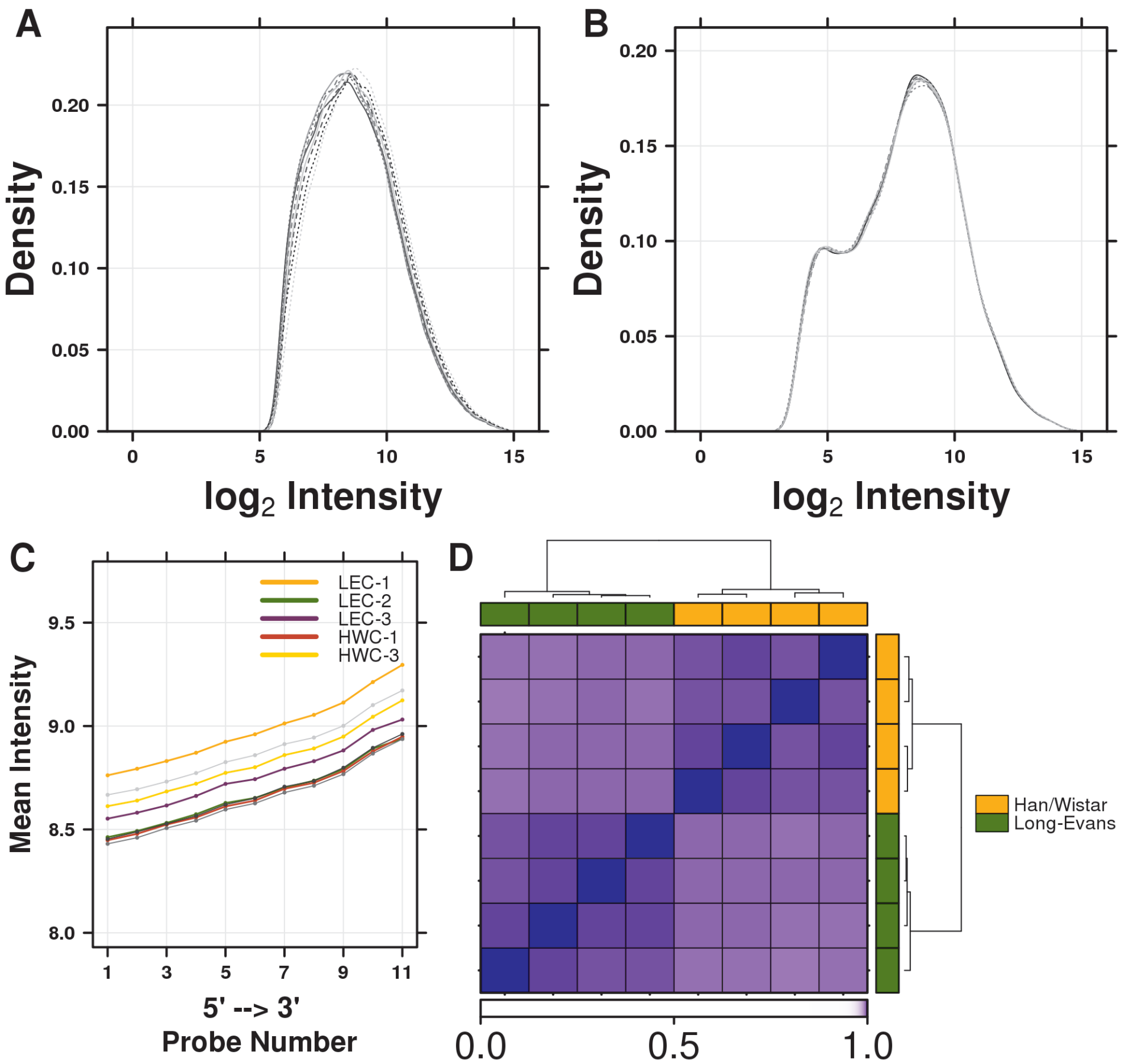

Supplement: Supplementary file 5 — Additional file 5: Figure S5: Data Quality Assessment: Rat, Hypothalamus. Comparison of distributions of probe-level log2 intensities before (A) and after (B) RMA normalization. The average intensities of probes across ProbeSets were examined using an RNA degradation plot (C). Intra-array correlation was assessed with a heatmap generated using complete agglomerative clustering, with Pearson’s coefficient employed as the similarity metric (D). (PDF 403 KB) [file 12864_2014_6766_MOESM5_ESM.pdf]

# Data Quality Assessment

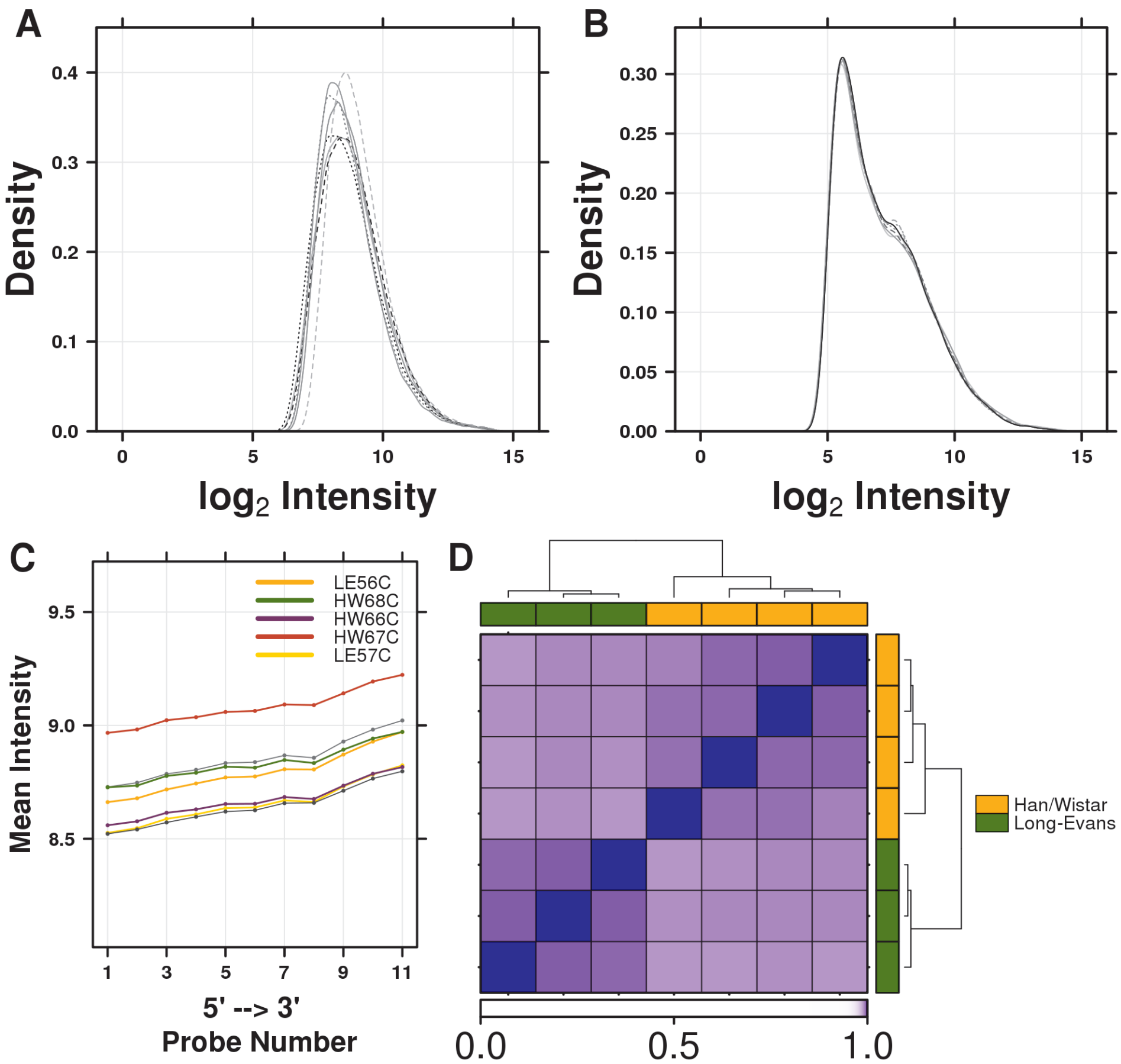

Supplement: Supplementary file 6 — Additional file 6: Figure S6: Data Quality Assessment: Rat, Adipose, Outlier Removed. Comparison of distributions of probe-level log2 intensities before (A) and after (B) RMA normalization with the outlier array removed (RAE2302_083106W_AO07.CEL). The average intensities of probes across ProbeSets were examined using an RNA degradation plot (C). Intra-array correlation was assessed with a heatmap generated using complete agglomerative clustering, with Pearson’s coefficient employed as the similarity metric (D). Removal of the outlier array improved overall spatial and distributional homogeneity. (PDF 400 KB) [file 12864_2014_6766_MOESM6_ESM.pdf]

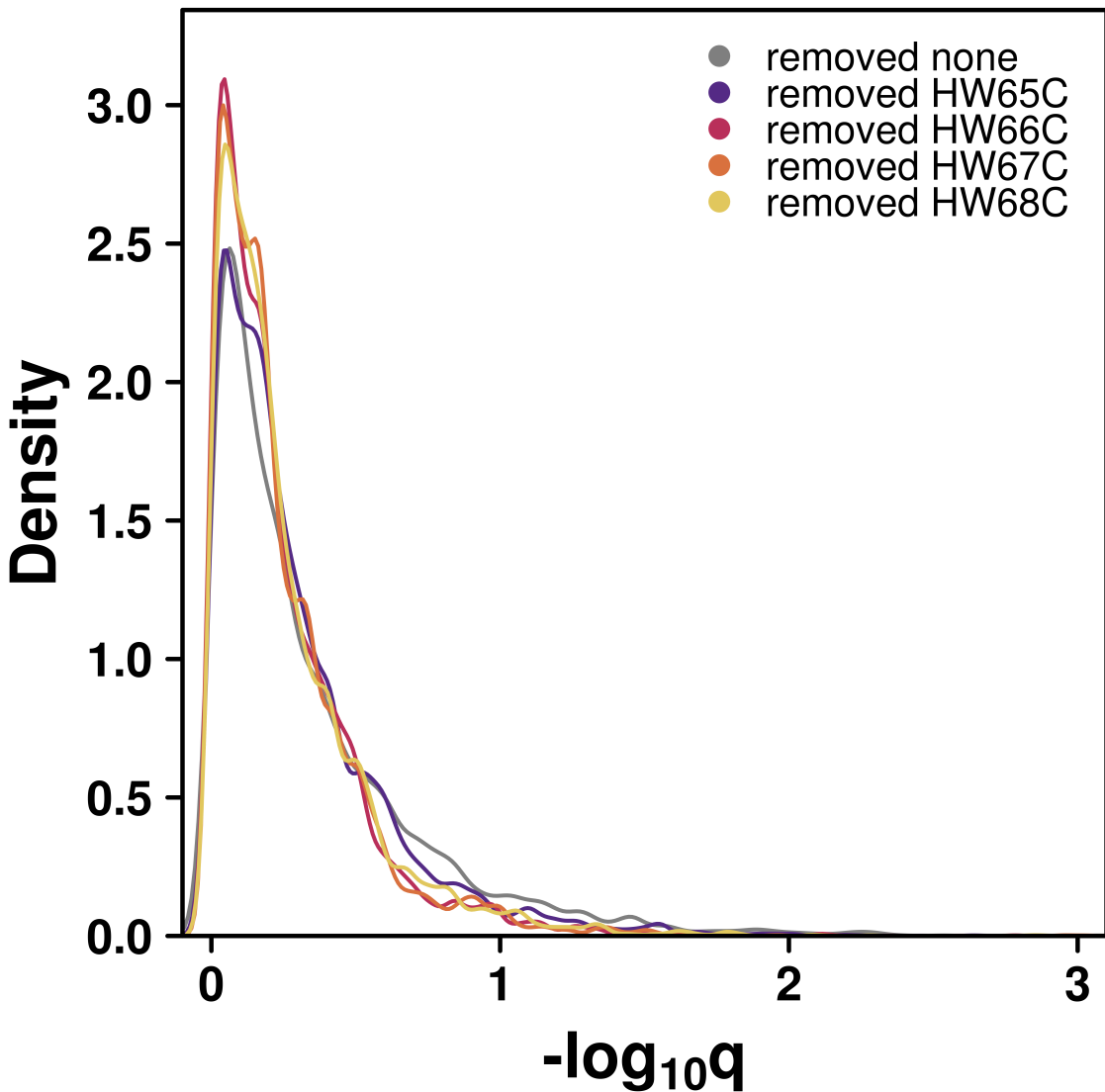

Supplement: Supplementary file 14 — Additional file 14: Figure S7: Differential Power Analysis: Rat, Adipose. To assess the effect of removing one L-E outlier (RAE2302_083106W_AO07.CEL) on statistical power, one array from the H/W group was systematically removed and the data re-normalized and re-fitted. Similar patterns of q-value densities were observed following each removal, justifying proceeding with subsequent analyses using all H/W arrays. (PDF 1 MB) [file 12864_2014_6766_MOESM14_ESM.pdf]

A

## Rat Liver

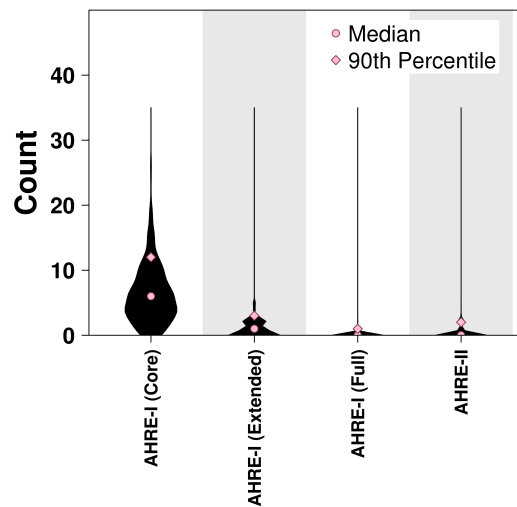

## Rat Adipose

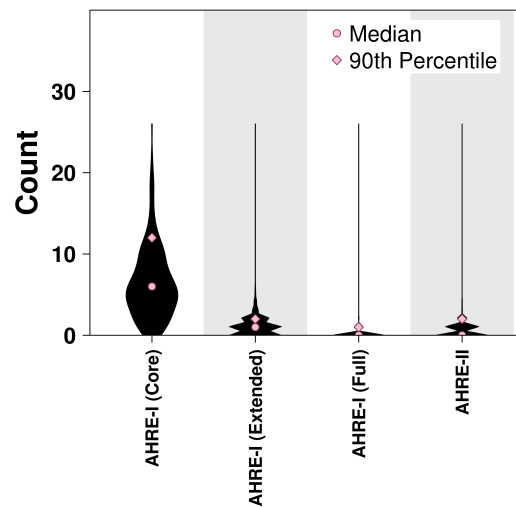

## Rat Hypothalamus

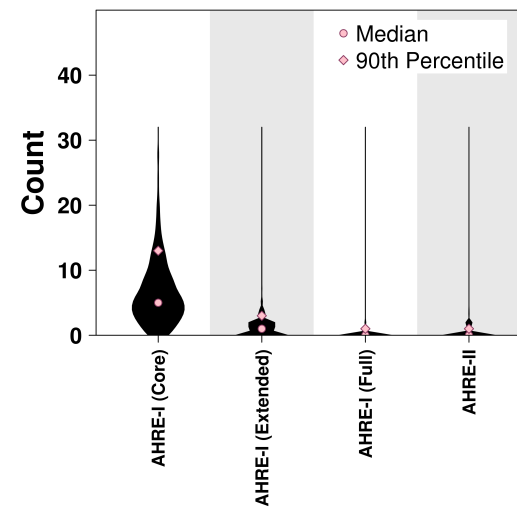

B

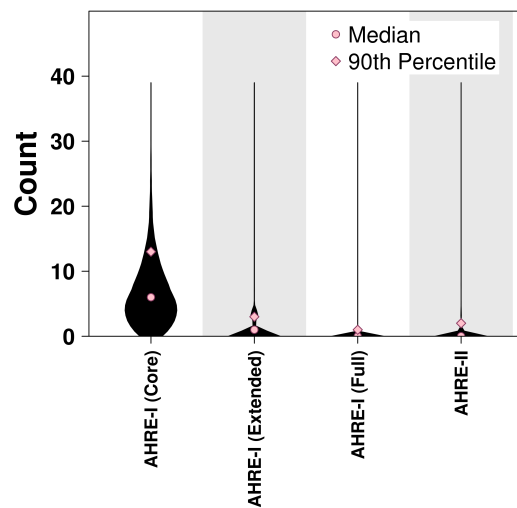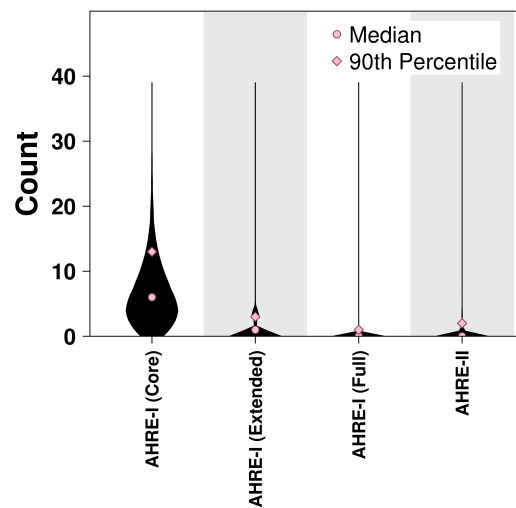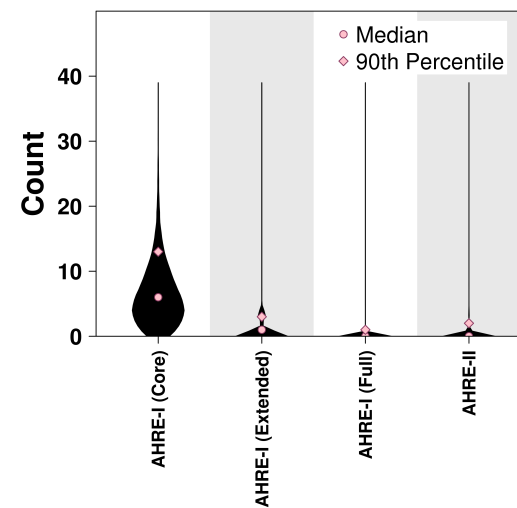

Supplement: Supplementary file 15 — Additional file 15: Figure S8: Transcription Factor Binding Analysis: Rats, Motif Counts. Kernel densities of counts for AHRE-I (Core), AHRE-I (Extended), AHRE-I (Full) and AHRE-II motifs are shown for significant (A) and non-significant (B) genes in rat tissues. The median is represented by the circular point while the 90th percentile is represented by the diamond point. (PDF 953 KB) [file 12864_2014_6766_MOESM15_ESM.pdf]

**A**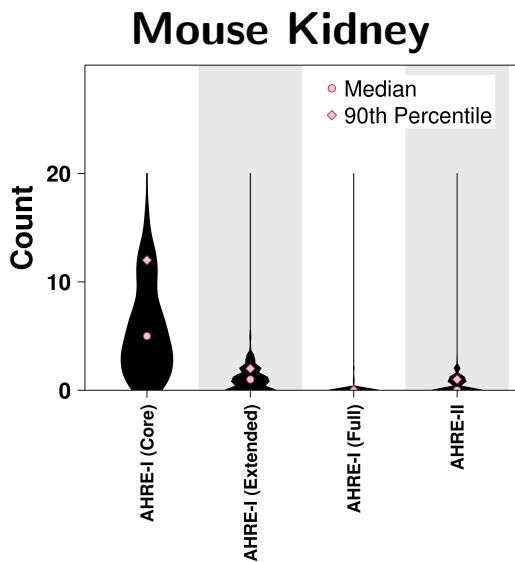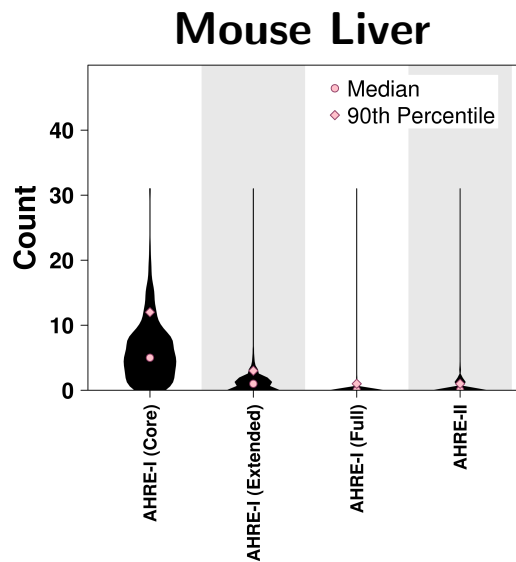**B**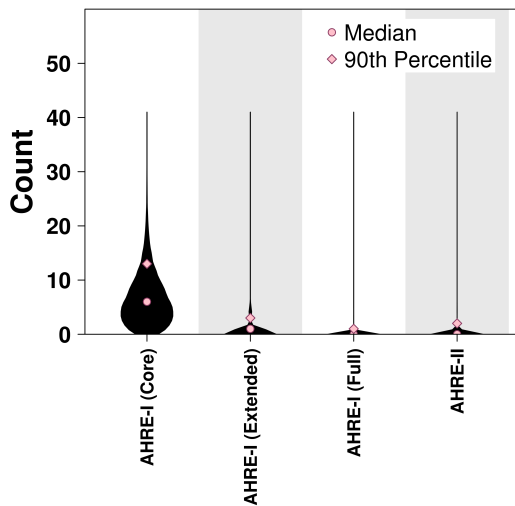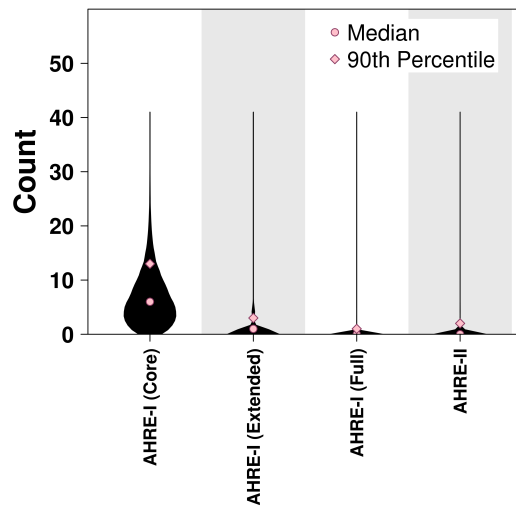

Supplement: Supplementary file 16 — Additional file 16: Figure S9: Transcription Factor Binding Analysis: Mice, Motif Counts. Kernel densities of counts for AHRE-I (Core), AHRE-I (Extended), AHRE-I (Full) and AHRE-II motifs are shown for significant (A) and non-significant (B) genes in mouse tissues. The median is represented by the circular point while the 90th percentile is represented by the diamond point. (PDF 638 KB) [file 12864_2014_6766_MOESM16_ESM.pdf]

**A****Rat Liver**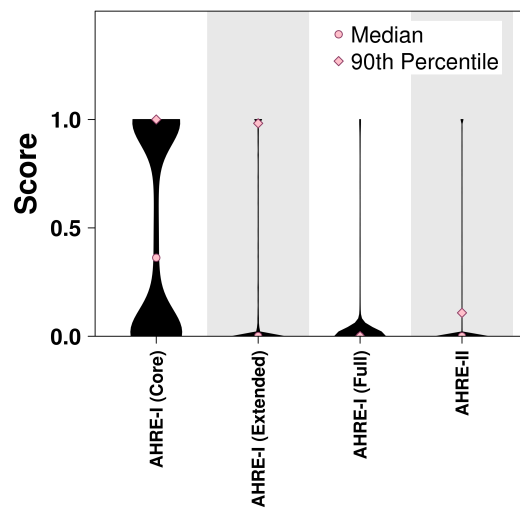**Rat Adipose**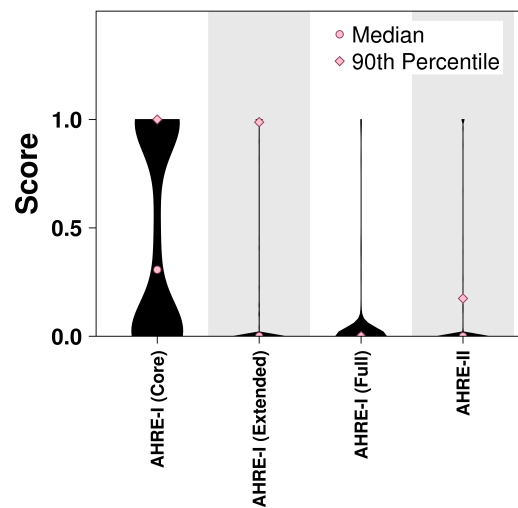**Rat Hypothalamus**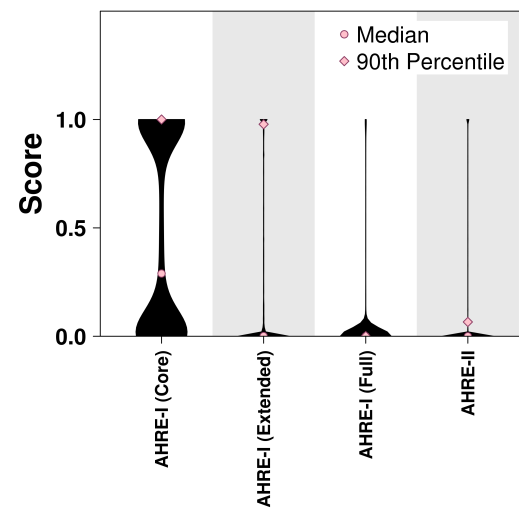**B**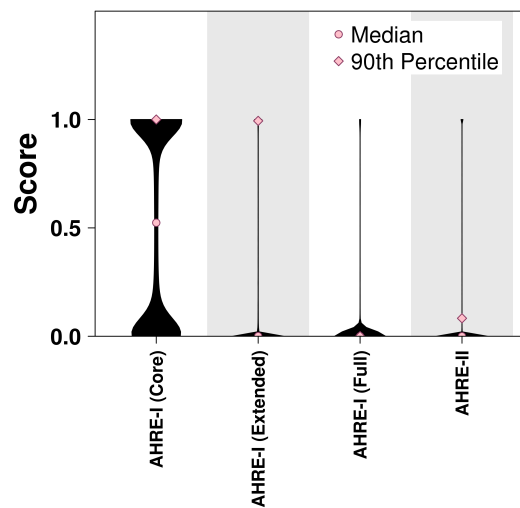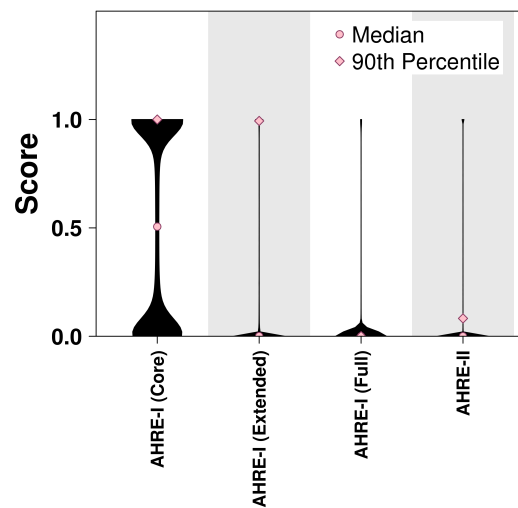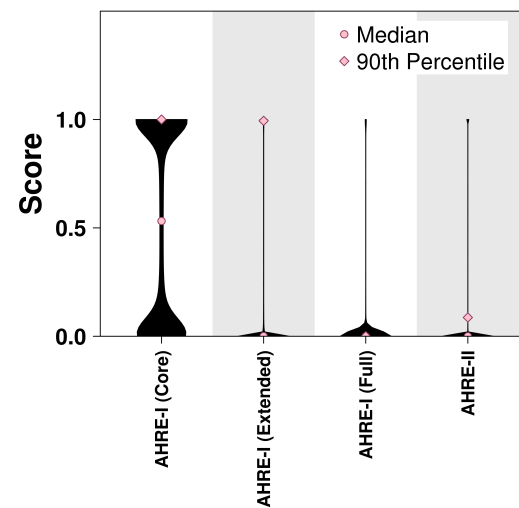

Supplement: Supplementary file 17 — Additional file 17: Figure S10: Transcription Factor Binding Analysis: Rats, Motif Scores. Kernel densities of scores for AHRE-I (Core), AHRE-I (Extended), AHRE-I (Full) and AHRE-II motifs are shown for significant (A) and non-significant (B) genes in rat tissues. The median is represented by the circular point while the 90th percentile is represented by the diamond point. (PDF 949 KB) [file 12864_2014_6766_MOESM17_ESM.pdf]

**A**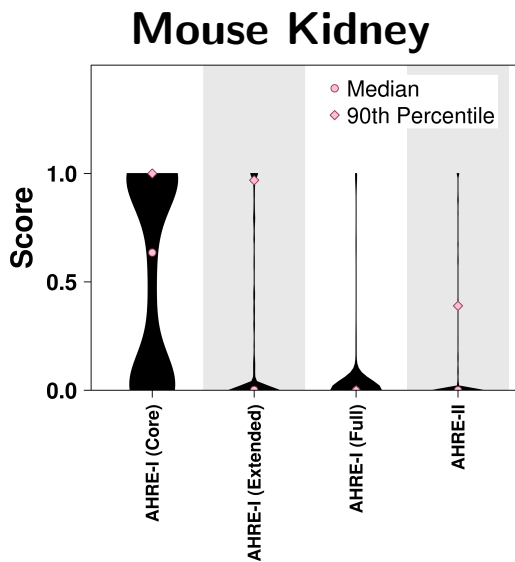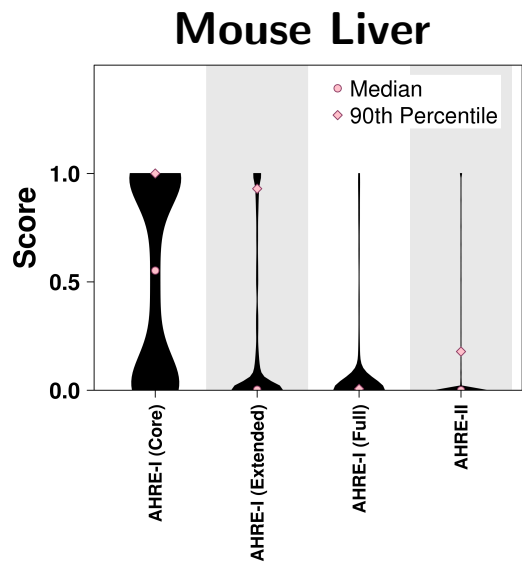**B**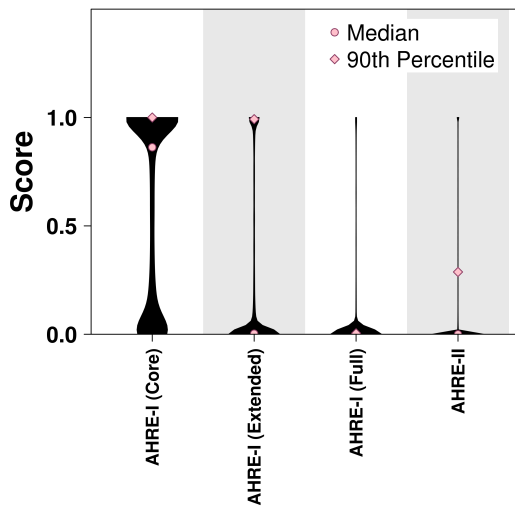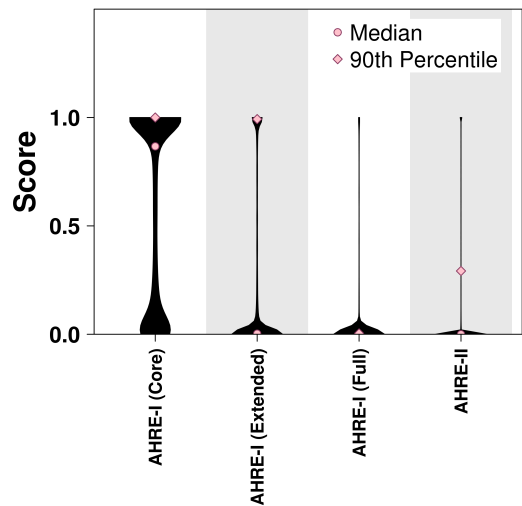

Supplement: Supplementary file 18 — Additional file 18: Figure S11: Transcription Factor Binding Analysis: Mice, Motif Scores. Kernel densities of scores for AHRE-I (Core), AHRE-I (Extended), AHRE-I (Full) and AHRE-II motifs are shown for significant (A) and non-significant (B) genes in mouse tissues. The median is represented by the circular point while the 90th percentile is represented by the diamond point. (PDF 656 KB) [file 12864_2014_6766_MOESM18_ESM.pdf]

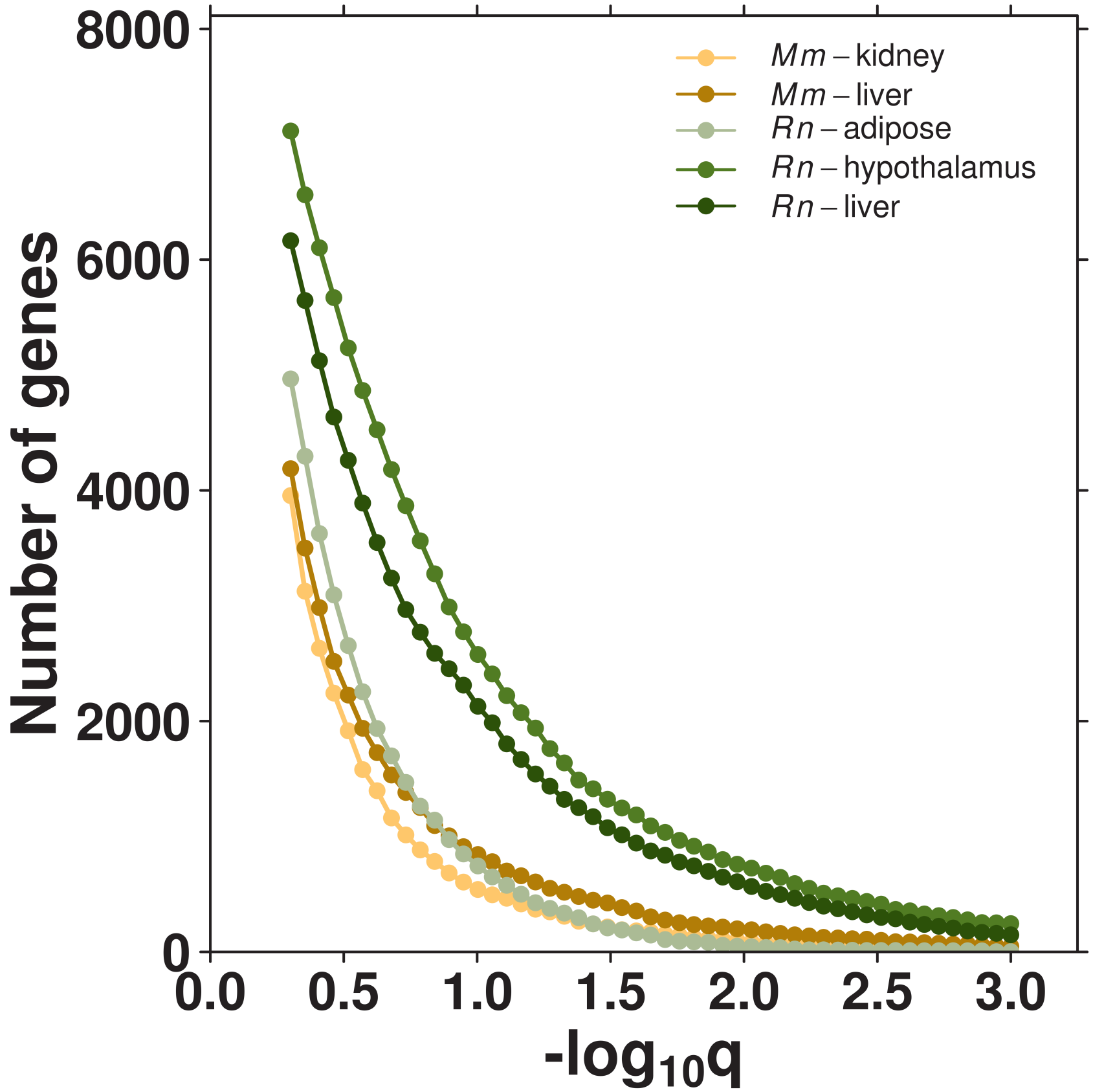

Supplement: Supplementary file 23 — Additional file 23: Figure S12: p-Value Sensitivity: count. The counts of significant genes after linear fitting and multiple testing correction were determined to be threshold-independent based on p-value sensitivity analysis. (PDF 1 MB) [file 12864_2014_6766_MOESM23_ESM.pdf]

**Fraction up-regulated**

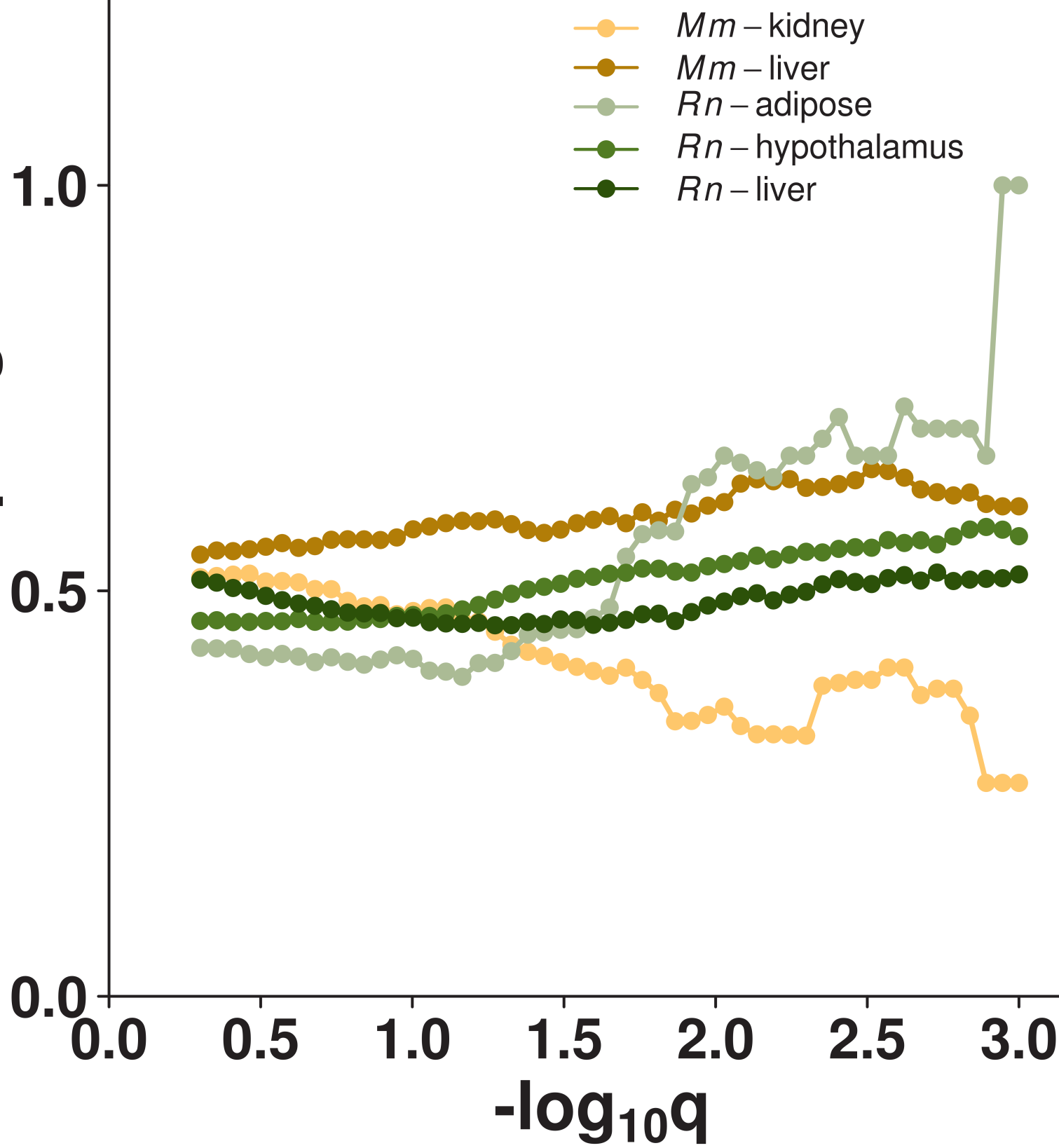

Supplement: Supplementary file 24 — Additional file 24: Figure S13: p-Value Sensitivity: direction. p-value sensitivity analysis revealed that the results were equally sensitive for detection of up- and down-regulated genes. (PDF 1 MB) [file 12864_2014_6766_MOESM24_ESM.pdf]

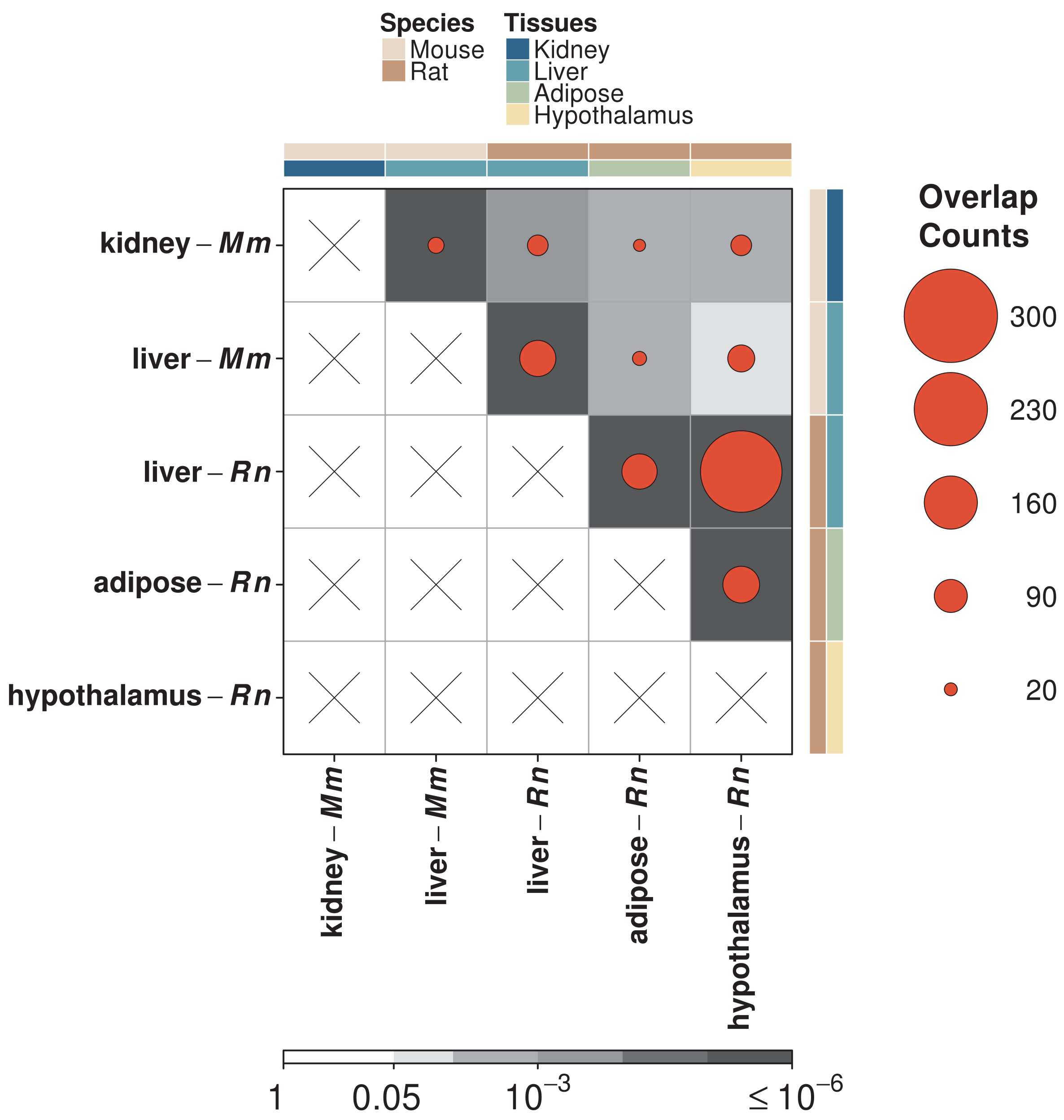

Supplement: Supplementary file 25 — Additional file 25: Figure S14: Raw Counts of Overlapping Genes. The counts of genes common to two tissues are shown for every tissue pair, with the magnitude of overlap represented by spot size and background shade denoting q-values calculated from hypergeometric testing. (PDF 655 KB) [file 12864_2014_6766_MOESM25_ESM.pdf]

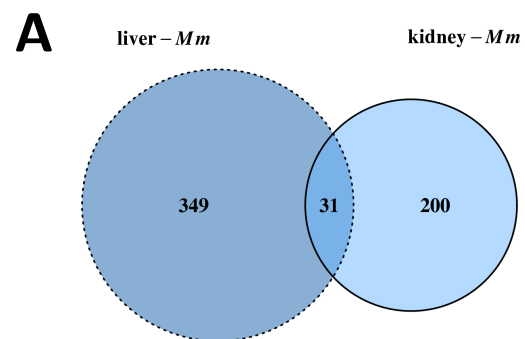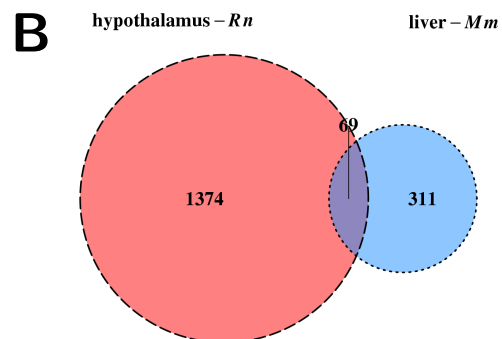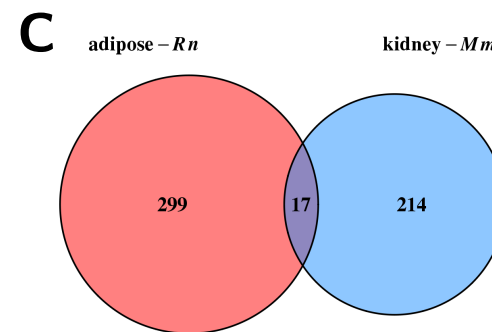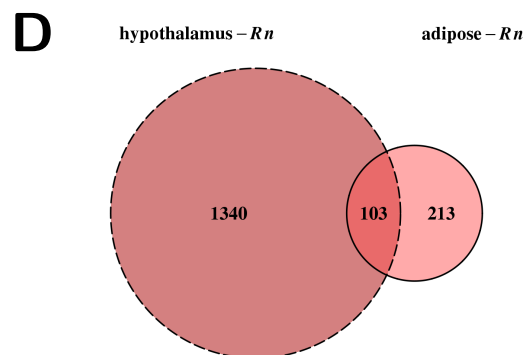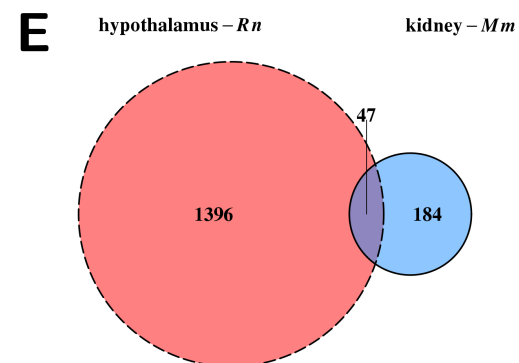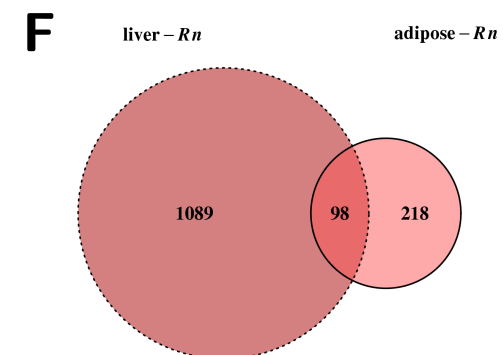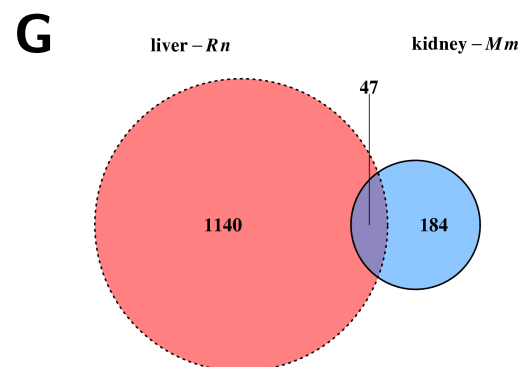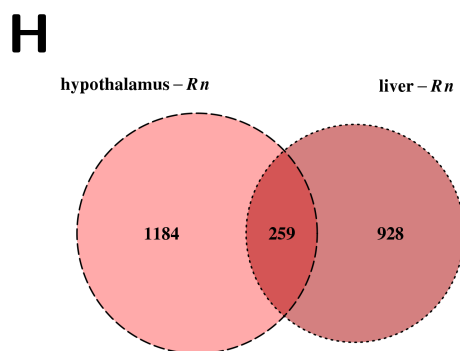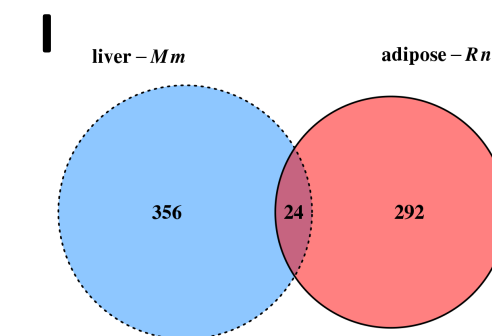

Supplement: Supplementary file 26 — Additional file 26: Figure S15: Gene Overlap Between Datasets. Overlapping genes are shown for mouse liver vs. mouse kidney (A), mouse liver vs. rat hypothalamus (B), mouse kidney vs. rat adipose (C), rat adipose vs. rat hypothalamus (D), mouse kidney vs. rat hypothalamus (E), rat adipose vs. rat liver (F), mouse kidney vs. rat liver (G), rat liver vs. rat hypothalamus (H) and mouse liver vs. rat adipose (I). Blue and red represent mouse and rat tissues respectively. Dotted, solid, dashed and no lines are used to visually differentiate liver, adipose, hypothalamus and kidney tissues. (PDF 639 KB) [file 12864_2014_6766_MOESM26_ESM.pdf]

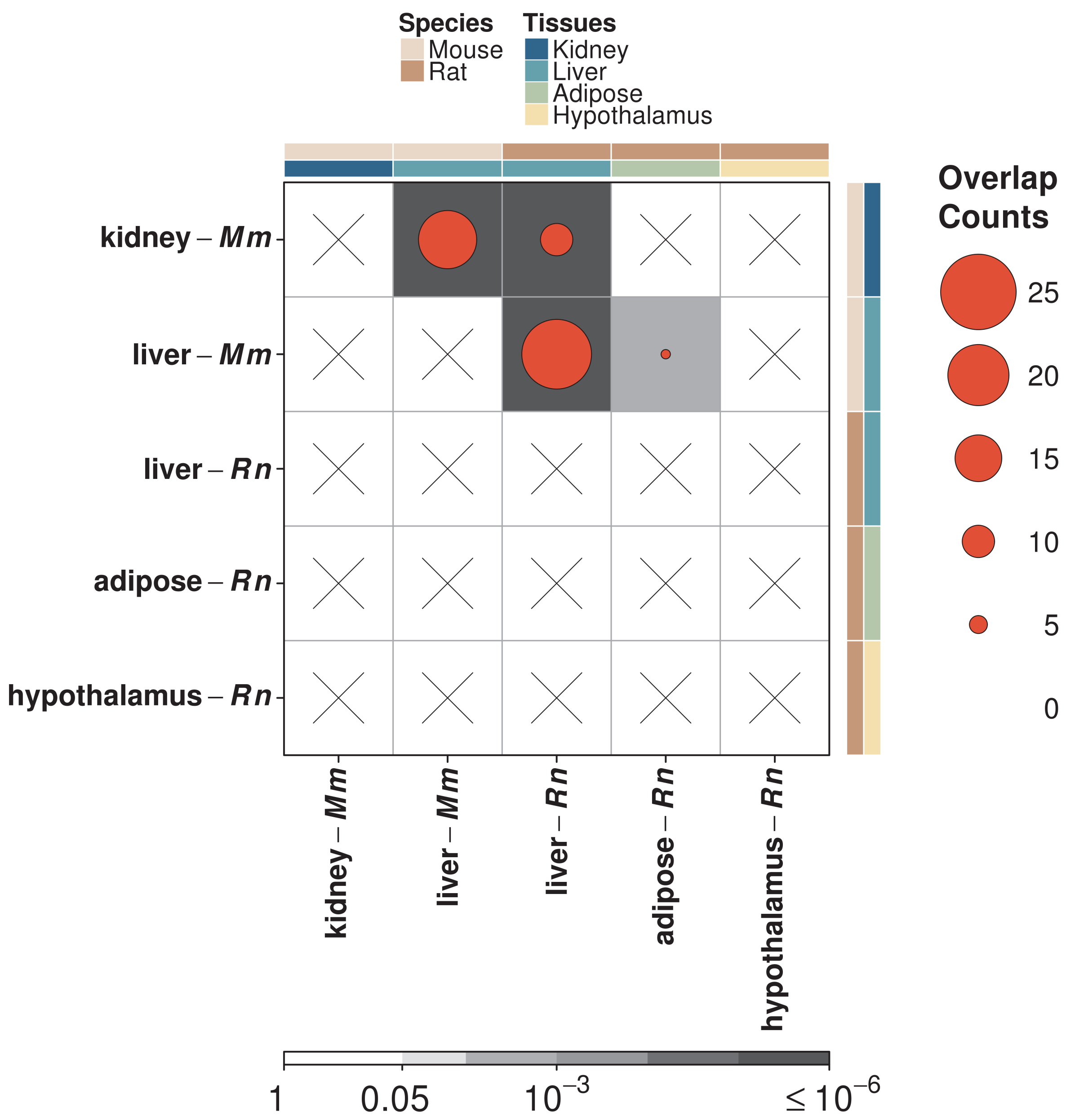

Supplement: Supplementary file 27 — Additional file 27: Figure S16: Raw Counts of Overlapping GO terms. The counts of GO terms common to two tissues are shown for every tissue pair, with the magnitude of overlap represented by spot size and background shade denoting q-values calculated from hypergeometric testing. (PDF 622 KB) [file 12864_2014_6766_MOESM27_ESM.pdf]

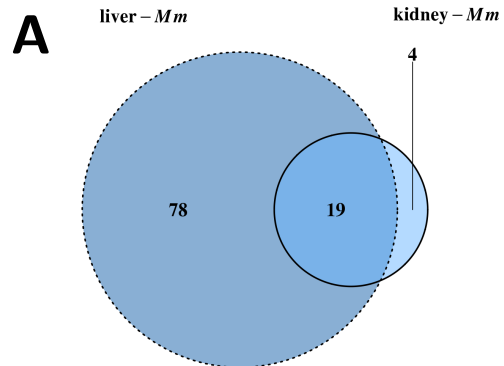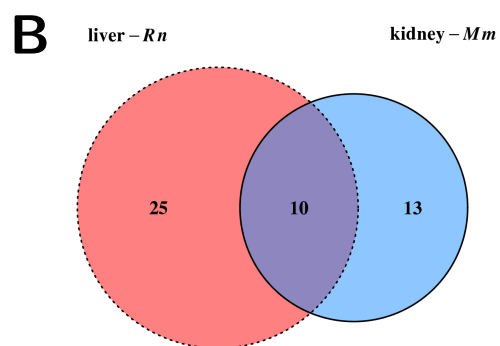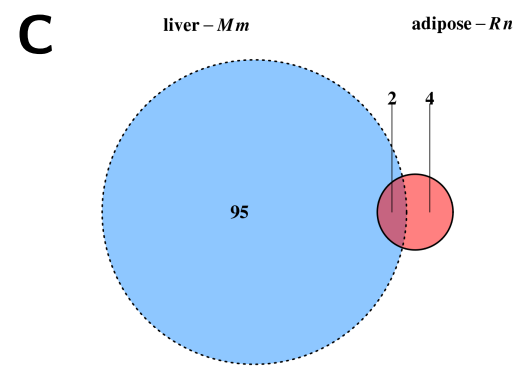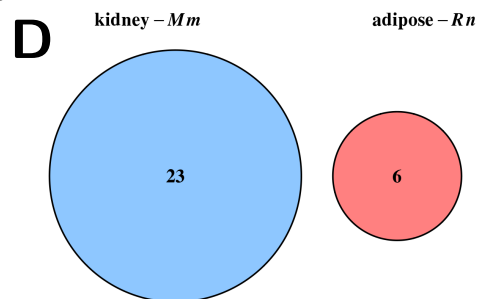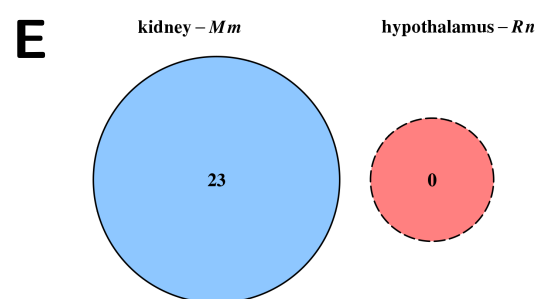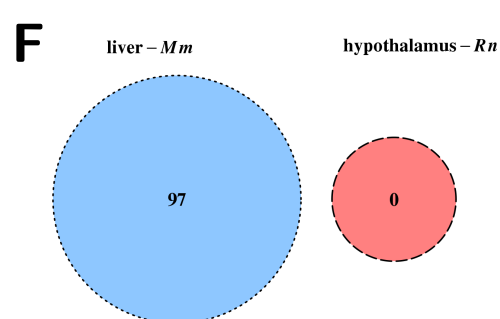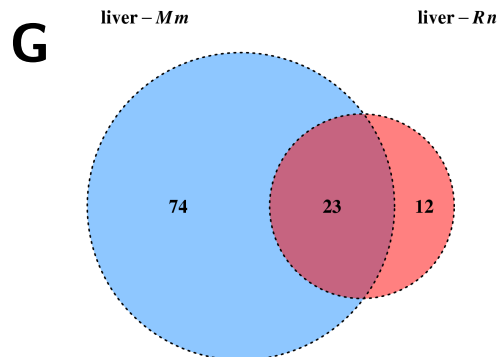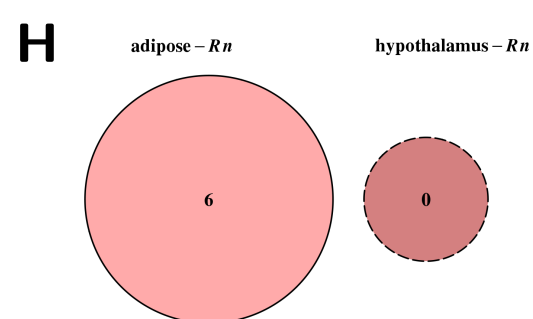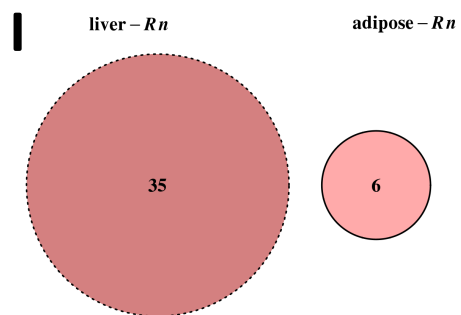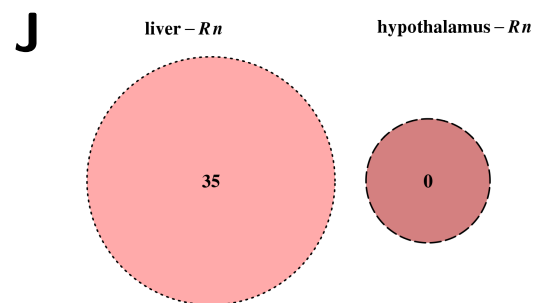

Supplement: Supplementary file 28 — Additional file 28: Figure S17: GO Term Overlap Between Datasets. Overlapping GO terms are shown for mouse liver vs. mouse kidney (A), mouse kidney vs. rat liver (B), mouse liver vs. rat adipose (C), mouse kidney vs. rat adipose (D), mouse kidney vs. rat hypothalamus (E), mouse liver vs. rat hypothalamus (F), mouse liver vs. rat liver (G), rat adipose vs. rat hypothalamus (H), rat liver vs. rat adipose (I) and rat liver vs. rat hypothalamus (J). Blue and red represent mouse and rat tissues respectively. Dotted, solid, dashed and no lines are used to visually differentiate liver, adipose, hypothalamus and kidney tissues. (PDF 646 KB) [file 12864_2014_6766_MOESM28_ESM.pdf]

**A**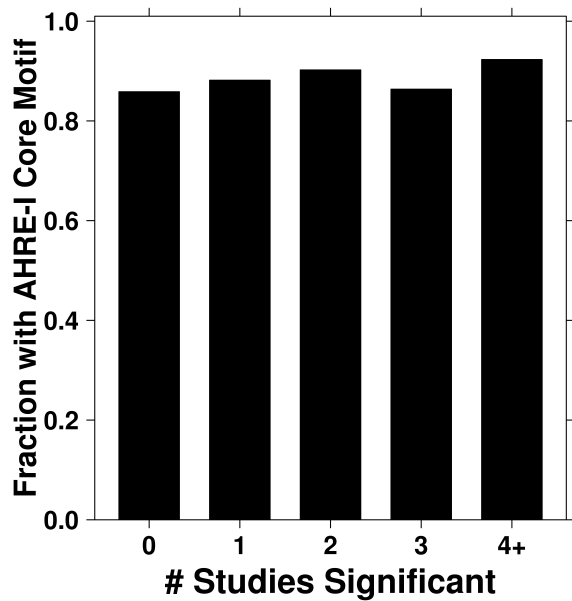**B**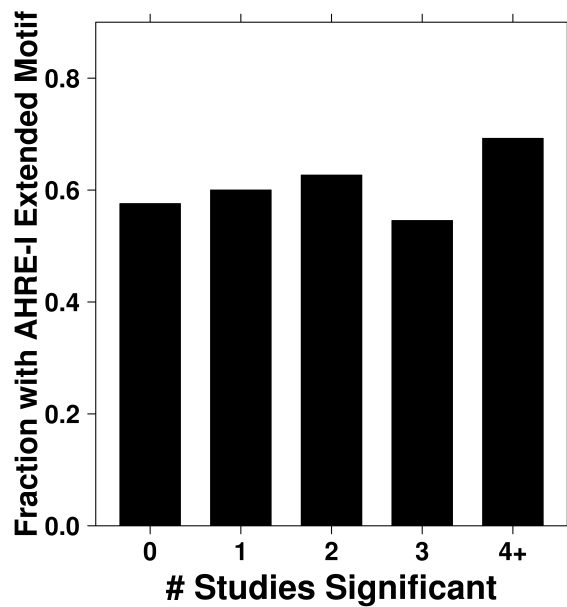**C**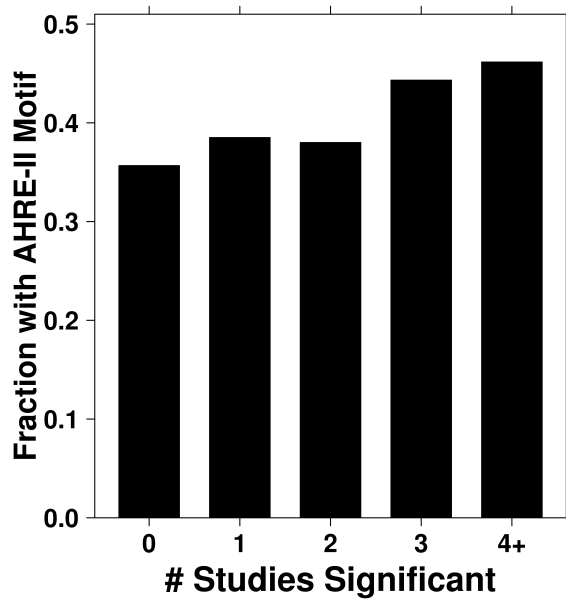

Supplement: Supplementary file 29 — Additional file 29: Figure S18: Fraction of genes with TFBS motifs. A comparison of genes differentially-abundant across multiple tissues and their fractions of observed AHRE-I (Core) (A), AHRE-I (Extended) (B) and AHRE-II (C) motifs. (PDF 876 KB) [file 12864_2014_6766_MOESM29_ESM.pdf]
