# Supplementary material for: Cross-species transcriptomic analysis elucidates constitutive aryl hydrocarbon receptor activity
Source: BMC Genomics. 2014 Dec 3;15(1):1053. doi: 10.1186/1471-2164-15-1053 (PMC4301818; doi:10.1186/1471-2164-15-1053)
Supplement: Supplementary file 13 — Additional file 13: R Code for Generating Bootstrap P-value. The R code used to generate the bootstrap p-value for the overlap between three rat tissues. (PDF 24 KB) [file 12864_2014_6766_MOESM13_ESM.pdf]

```r
### generate.bootstrap.p.value.R ###################################################################
# estimate probability of overlap of three datasets vs. expected by chance alone

### FUNCTIONS #######################################################################################
# function that carries out bootstrap p-value generation for 1 permutation
hypergeometric.data.generate <- function(list.of.sample.lengths, total.length=10445) {
	true.positions <- lapply(
		list.of.sample.lengths,
		function(sample.length) {
			return(
				sample(
					x = total.length,
					size = sample.length
					)
				);
			}
		);
	table.positions <- table(unlist(true.positions));
	common.positions <- table.positions[table.positions==3];
	return(length(common.positions));
	}

### MAIN ############################################################################################
# read in main results dataframe
results.dataframe <- read.table(
	file = '2014-08-07_Constitutive.AHR_common.annotated.fit.tissues.txt',
	header = TRUE,
	sep = "\t",
	quote = "",
	comment.char = "",
	fill = TRUE
	);

# clean up dataframe to only keep the columns needed for this analysis
subset.results <- results.dataframe[, grep('Q.|HID', names(results.dataframe))];

### SET LENGTHS #####################################################################################
# establish lists of significant genes
rat.liver.sig.genes <- subset.results$HID[
	subset.results[grep('Rat.Liver', names(subset.results))] < 0.05
	];
rat.adipose.sig.genes <- subset.results$HID[
	subset.results[grep('Rat.Adipose', names(subset.results))] < 0.05
	];
rat.hypothalamus.sig.genes <- subset.results$HID[
	subset.results[grep('Rat.Hypothalamus', names(subset.results))] < 0.05
	];

# count how many overlap in all three datasets
observed.overlap <- length(
	intersect(
		intersect(
			rat.liver.sig.genes,
			rat.adipose.sig.genes
			),
		rat.hypothalamus.sig.genes
		)
	);

# initiate list of genes
gene.lengths <- list(
	length(rat.liver.sig.genes),
	length(rat.adipose.sig.genes),
	length(rat.hypothalamus.sig.genes)
	);

# set number of permutations
permutations <- 1000000;

# apply bootstrap, hypergeometric mimic:
```

```
#       find occurences of overlap counts >= observed count
counts.of.overlaps <- replicate(
        n = permutations,
        expr = hypergeometric.data.generate(gene.lengths)
        );
frequency.greater <- length(
        which(counts.of.overlaps >= observed.overlap)
        ); # 0 occurrences of overlap > 28, highest is 9

# estimated p-value from permutations
p.value.estimate <- frequency.greater/ permutations;
print(p.value.estimate);

# Result after running code:
#       p.value.estimate = 0
#       therefore, p < 1/1,000,000
```
